# Age-specific associations between perceptions of community environmental quality, restorative environmental perception and community identity: an interpretative perspective based on the Homo Urbanicus

**DOI:** 10.3389/fpsyg.2026.1961297

**Published:** 2026-10-29

**Authors:** Chenda Guo, Lujie Liu, Jiale Fei, Liusha Chen, Qiwei Zhu

**Affiliations:** University of Shanghai for Science and Technology, Shanghai, China

**Keywords:** age-differentiated associations, community identity, Homo Urbanicus theory, perceived community environmental quality, restorative environmental perception

## Abstract

**Context:**

Understanding the associations between residents’ perceived community environmental quality and community identity, and their age-related differences, is important for advancing all-age-friendly communities.

**Methods:**

Drawing on Hok-Lin Leung’s Homo Urbanicus theory, this study analyzed 929 questionnaires collected in 2025 from 36 residential communities in Yangpu District, Shanghai. Structural equation modelling was used to examine the association structure among perceived community environmental quality, perceived restorative environment and community identity, and to test whether the three structural paths differ across four age groups (children, young adults, middle-aged and older adults), using measurement invariance tests, cross-group equality constraints and paired parameter comparisons, with cluster-robust standard errors.

**Results:**

(1) Perceived restorative environment significantly mediated the association between perceived community environmental quality and community identity: the indirect effect accounted for 51.5% of the total effect and the direct effect remained significant, the two links being comparable in magnitude. (2) Measurement invariance was broadly supported, permitting cross-group comparison of the structural paths, and the mediation held in all four groups (bias-corrected bootstrap, 5,000 resamples). Cross-group differences were localized: the path from perceived restorative environment to community identity was stronger in the middle-aged than in the older group (cluster-robust difference = 0.270, *p* = 0.021; significant under all three specifications). The children’s group scored numerically higher on the path from perceived community environmental quality to perceived restorative environment, but this did not consistently reach significance and is reported descriptively. Direct effects did not differ significantly across the four groups (Δχ^2^ = 2.670, Δdf = 3, *p* = 0.445), so the non-significant direct effect in the children’s group is not interpreted as an absent effect.

**Discussion:**

These findings are associations derived from cross-sectional data, not causal effects or observed psychological transformation. Within the ‘events, people, time and space’ framework of the Homo Urbanicus theory, life-stage differences in the weighting of self-existence and coexistence needs offer a coherent interpretation of the age-specific path patterns. The results support the differentiated provision of restorative space in age-friendly community planning.

## 1 Introduction

As China’s urbanization process shifts from incremental expansion to the optimization of existing stock (Li et al., 2021), the environmental quality of communities—as the core spatial units of urban residents’ daily lives and interpersonal interactions (Yang, 2007; Gui and Huang, 2006)—is attracting increasing attention from both academia and policymakers for its association with residents’ mental health and sense of belonging (Jin et al., 2017; Jiang and Sun, 2022; Leslie and Cerin, 2008; Kim and Kim, 2017; Reuben et al., 2022; Liu et al., 2025). Communities are not merely the physical vessels of residential functions, but also crucial settings for residents’ daily interactions, emotional attachment and the formation of identity (Wang, 2018; Yan and Ye, 2019; Xin and Ling, 2015; Zhang et al., 2017; Bonaiuto et al., 1999; Fan, 2022; Scannell and Gifford, 2010). The United Nations Sustainable Development Goal 3 (SDG 3) emphasizes health and well-being for all ages (United Nations, 2017; Yu, 2024). As the living space closest to residents, whether the perceived environmental quality of a community meets the psychological recovery needs of residents across different age groups—as well as the relationship between this perception and a sense of community belonging, and the age-related differences therein—is directly linked to the grassroots implementation of this goal.

Research into the relationship between the community environment and residents’ psychological well-being has yielded a wealth of empirical evidence. Yang (1997), using different types of residential areas in the Shanghai metropolitan area as case studies, proposed that the quality of the community environment constitutes the community’s ‘support and security system’, comprising two core dimensions: the standard of physical facilities and the vitality of social networks. They systematically demonstrated the bidirectional interactive relationship between environmental quality and residents’ willingness to settle in the area, as well as their sense of belonging to the community. Subsequently, scholars have deepened research in this field from various perspectives: at the built environment level, factors such as community greening, public facilities, environmental hygiene, safety management and property services have been shown to be closely related to residents’ psychological states and behavioral patterns (Yue et al., 2022; Yang et al., 2019; Han et al., 2024; Peng and Wang, 2024; Mou et al., 2025); at the social environment level, community cohesion and neighborly trust have been identified as key mediating mechanisms linking environmental improvements to residents’ subjective well-being (Yang and Zhang, 2022; Li, 2024; Luo, 2023; Lee and Jeong, 2021). However, existing research has largely focused on single age groups or individual environmental factors, with a lack of studies systematically examining the complete chain mechanism of ‘perceived community environmental quality → psychological perception → community identity’ from an all-age perspective.

The theory of restorative environments offers an important perspective for understanding the transformative processes between the environment and the psyche. The theory of restorative environments, proposed by Kaplan and his wife, suggests that environments possessing the four characteristics of ‘being away’, ‘fascination’, ‘extent’ and ‘compatibility’ can effectively alleviate psychological stress and restore attentional resources (Kaplan, 1995; Laumann et al., 2001; Su and Xin, 2010; Shao and Jin, 2025; McDonnell and Strayer, 2024). Existing research has confirmed that restorative experiences can significantly and positively predict an individual’s attachment to a place (Feng and Cui, 2017; Ratcliffe and Korpela, 2016; Liu et al., 2020; Yoon et al., 2023; Korpela and Hartig, 1996); in community settings, the higher the perceived quality of the restorative environment among residents, the more pronounced the effects on emotional relief and attentional recovery (Xi et al., 2021). However, existing research on restorative environments has largely focused on the psychological benefits of standalone open spaces such as parks and green spaces (Zhou et al., 2025), with few studies placing the perception of restorative environments qualities within the broader framework of the community environment to explore its mediating role between residents’ perceived community environmental quality and their sense of community identity. The mechanism by which a community, through the psychological pathway of providing restorative experiences, transforms residents’ perceived environmental quality into a subjective sense of emotional belonging has not yet been fully examined (Wang et al., 2025).

Individual factors play a significant moderating role in the processes of environmental perception and psychological transformation. Research indicates that there are significant differences in the needs, preferences and usage patterns of community environments across different age groups (Li, 2019; Wang and Chang, 2021; Huang et al., 2024): children place greater emphasis on the safety and enjoyment of activity spaces; young adults value the comfort, aesthetic appeal and recreational and social functions of the environment; middle-aged adults prioritize community safety, convenience and neighborly support; whilst older adults place a high degree of reliance on the accessibility, tranquillity and social atmosphere of the community environment. With regard to restorative environmental perception, relevant research indicates that the accessibility, safety, openness and familiarity of community spaces play a key role in the perception of restorative environments amongst older adults. However, few studies have treated age as a moderator variable to systematically examine the age-specific pathways linking the perceived quality of the community environment to community identity across the entire age spectrum.

To explain this complex, age-dependent psychological transformation process, a theoretical framework is required that can simultaneously account for individual differences, environmental characteristics and psychological mechanisms. Homo Urbanicus theory proposed by Professor Hok-Lin Leung provides a systematic analytical approach for this very purpose. This theory defines an ‘urban dweller’ as ‘an individual who rationally chooses to live in a community in order to pursue opportunities for spatial contact’ (Liang, 2012), emphasizing that urban residents (typical urban dwellers) actively seek such opportunities through rational settlement, thereby pursuing a dynamic balance between self-existence and coexistence (Liang, 2014). As a unit of human habitation (typical human settlement), a community’s environmental quality, spatial experience and social interaction serve as key factors underpinning residents’ choices, adaptation and sense of belonging. In this context, ‘self-existence’ refers to the fulfilment of an individual’s basic needs for safety, comfort and recuperation, whilst ‘coexistence’ refers to the identity with collective values such as a sense of belonging to the community and emotional connections.

Unlike the ‘rational economic agent’ in economics, which posits humans as isolated individuals seeking solely to maximize their own interests, Homo Urbanicus theory is based on a fundamentally different assumption regarding human nature. Professor Hok-Lin Leung points out that what makes us human is that our nature encompasses two dual aspects: self-existence and coexistence with others (coexistence). These two aspects are not in a zero-sum, opposing relationship, but rather constitute the primary principle of simultaneous and equal importance, mutually reinforcing one another. This implies that in communal living, people not only focus on their own safety, comfort and well-being, but also naturally seek a sense of belonging within their community and emotional connections with others.

However, whilst existing research has confirmed a positive correlation between the perceived quality of the community environment and residents’ mental health and sense of belonging, it often reduces residents to statistical units categorized by age, merely describing differences in the levels of identity among various groups without explaining why the mechanisms underlying these age-related differences vary; A deeper issue lies in the fact that, even where some studies recognize that people cannot be simply reduced to symbols, their assumptions about human nature remain constrained by the ‘rational economic agent’ framework. This treats residents as isolated individuals who make cost–benefit calculations based solely on the advantages and disadvantages of their environment, overlooking the dual aspects of human nature—the need for both self-existence and coexistence; The limitations of this assumption make it difficult for existing research to truly reveal the essence of age-related differences, as these differences are not merely statistical categorization variables, but rather a manifestation of structural shifts in the relative weighting of self-existence and coexistence needs as one progresses through the stages of life.

A further strength of the ‘Homo Urbanicus’ theory lies in its integration of different life stages (people), varying spatial interaction needs (activities), differing durations of residence (time) and scales of activity (space) into a unified analytical framework. This provides an analytical tool that is both practical and theoretically profound for examining how the community environment relates to community identity among different age groups through restorative experiences. Consequently, this study adopts the ‘Homo Urbanicus’ theory with the aim of systematically explaining the structural relationships—and their age-related variations—between the perceived quality of the community environment, perceptions of restorative environments, and community identity, from the perspectives of rational settlement, positive interaction, the alignment of subjective and objective factors, and the balance between self-sufficiency and coexistence.

Homo Urbanicus theory offers a more thorough explanatory framework for this: the balance between self-existence and coexistence is in itself an objective tendency that can be identified and guided; it is not a by-product of the pursuit of individual self-interest, but rather the natural manifestation in space of the dual aspects inherent in human nature. This shift in the underlying assumption regarding human nature advances the research perspective from the functional level of ‘whether environmental provision meets demand’ to the deeper psychological level of ‘how environmental quality is transformed into emotional attachment through psychological perception’. Consequently, the core proposition of community planning is redefined: it is no longer about acting as a mediator amidst conflicting interests, but rather about how, through the differentiated allocation of spatial resources, to provide residents of different ages and at different life stages with positive opportunities for spatial contact that align with their structural needs for self-existence and coexistence, thereby enabling residents to naturally achieve a balance between self-existence and coexistence in their daily communal lives (Liang, 2014). This shift in perspective distinguishes the present study from existing literature not only through the introduction of a new theoretical framework, but also by elevating the discussion of age differences from statistical group comparisons to an explanatory level concerning the evolution of human needs structures; this constitutes the core contribution of this study.

### 1.1 Research questions

This study aims to answer the following research questions:

1. What are the current characteristics of perceived community environmental quality, restorative environmental perception and community identity?
2. Is there a significant positive correlation between perceived community environmental quality and restorative environmental perception? Does restorative environmental perception act as a mediating variable in the relationship between perceived community environmental quality and community identity?
3. Do the strengths of the three structural paths—perceived community environmental quality → restorative environmental perception, restorative environmental perception → community identity, and perceived community environmental quality → community identity—differ across age groups (children, young adults, middle-aged adults and older adults)?

## 2 Theoretical framework, research methods and measurement

### 2.1 Theoretical framework

The Homo Urbanicus theory is an urban planning theory proposed by the Canadian planning scholar Professor Hok-Lin Leung. It emphasizes that humans seek ‘opportunities for spatial contact’ through rational settlement; its core lies in matching the dynamic relationship between the ‘typical urban dweller’ and the ‘typical human settlement’, thereby establishing a balance between self-existence and coexistence (Liang, 2012; Liang, 2014; Liang, 2020).

Within the theoretical framework of the ‘Homo Urbanicus’, the human settlement environment is the result of the interplay and dynamic changes among the four elements of ‘events, people, time and space’ (hereinafter referred to as the ‘four elements’) (Guo and Zhou, 2020). Among these, ‘people’ refers to basic attributes such as age, gender and life stage, as well as contingent attributes such as economic and social status; ‘events’ refers to the opportunities for spatial engagement sought by ‘urban dwellers’; ‘time’ refers to the timing at which the individual is situated; and ‘space’ refers to the spatial scope of focus. These four elements collectively define the identity and needs structure of the ‘typical urban dweller’ within a specific living environment, providing a unified theoretical tool for analyzing differences in environmental perception and behavior amongst different resident groups. It should be noted that the core variable of this study, ‘restorative environmental perception’, is not directly equivalent to ‘events’, but rather represents residents’ subjective experiences and evaluations of ‘events’ (opportunities for spatial engagement). In other words, the spatial contact opportunities (events) provided by the community, once perceived by residents, give rise to restorative experiences (restorative environmental perception), which are linked to the formation of community identity—this is the theoretical expectation, the actual direction of which remains to be empirically tested.

As the basic unit of urban living, the community is, in essence, the core setting in which ‘urban dwellers’ rationally select opportunities for spatial interaction, guided by the principle of balancing self-existence and coexistence. Opportunities for spatial interaction are divided into positive and negative categories: positive opportunities are spatial interactions actively sought by residents that bring comfort, relaxation, pleasure and social engagement; negative opportunities, on the other hand, are interactive scenarios within the residential environment that may give rise to stress, alienation, conflict or exclusion. By providing positive opportunities for spatial contact—such as restorative experiences, friendly neighborly interactions, a safe and comfortable environment, and convenient services—the community serves as a key mediator linking individual needs with community life. Based on Doxiadis’s ‘science of human habitation’, living spaces require optimization through performance indicators—that is, replacing single-function zoning with quantifiable standards such as safety, comfort, convenience and aesthetic appeal (Liang, 2014; Wei et al., 2024)—and achieving a flexible allocation of residential resources through a multidimensional assessment of spatial contact opportunities. By treating the community as a typical human habitat and residents as typical urban dwellers, and by analyzing the ‘self-existence–coexistence’ balance between the two, an explanatory perspective can be provided for understanding the formation of community identity: environmental quality and spatial preferences correspond to the objective performance of the typical human habitat, whilst life satisfaction and emotional belonging reflect the subjective utility perceptions of urban dwellers.

The key to spatial opportunities lies in accessibility, whilst scale matching focuses on carrying capacity (Liang, 2014). As nodes of human habitation, communities must provide positive opportunities for interaction that strike an appropriate balance between accessibility and carrying capacity, thereby meeting residents’ rational needs for safety, comfort, social interaction and a sense of belonging. By systematically optimizing the performance of the living environment and fostering interaction between subjective and objective factors, an optimal pathway for community regeneration can be established, ultimately strengthening residents’ sense of belonging and identity with the community within a dynamic balance of self-existence and coexistence.

Based on the above analysis, the theoretical framework diagram for this study is shown in Figure 1.

**Figure 1:**
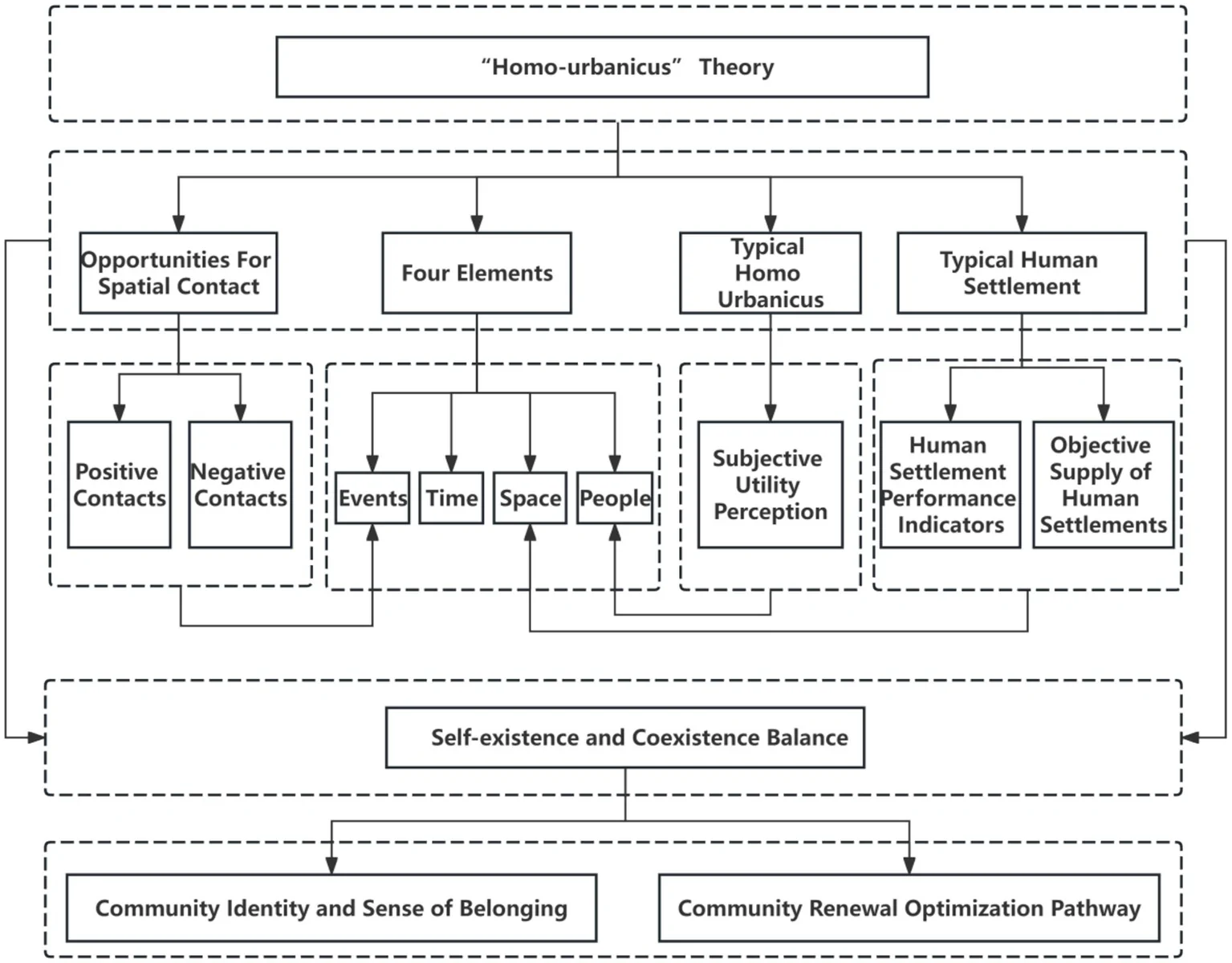
Theoretical framework diagram.

Based on the theoretical logic outlined above, this study proposes the following hypotheses (Table 1):

*H1*: There is a significant positive correlation between the perceived quality of the community environment and the perception of a restorative environment.

As a ‘typical living environment’, a community’s objective characteristics—such as green spaces, facilities and safety—form the fundamental conditions for residents to gain ‘opportunities for positive spatial engagement’. The higher residents’ evaluation of these living conditions, the more statistically significant the correlation between this evaluation and a stronger sense of relief from stress and the experience of restoring one’s attention; in other words, the higher the degree to which ‘self-existence needs’ are met, the stronger the perception of a restorative environment.

*H2*: Restorative environmental perception is significantly positively correlated with community identity.

Restorative experiences constitute a positive psychological benefit. Existing research indicates that restorative experiences are significantly positively correlated with place attachment. Accordingly, it is anticipated that residents reporting more restorative experiences will exhibit higher levels of community identity—within the explanatory framework of the ‘Homo Urbanicus’ theory, this expectation corresponds to the link between the fulfilment of self-existence needs and a sense of belonging to the community.

*H3*: The strength of the associations in the aforementioned pathways varies across age groups.

‘Typical urban dwellers’ of different age groups exhibit differences in the prioritization of self-existence versus coexistence needs, as well as in spatial usage habits; this leads to inter-group differences in the strength of the effect that environmental quality has on restorative environmental perception and community identity.

**Table 1:** Correspondence between theoretical concepts and hypothesis pathways.

Theoretical Concept	Operational Variable	Corresponding Hypothesis	Theoretical Explanation
Perceptual evaluation of the objective characteristics of typical living environments (safety, comfort, convenience and aesthetic appeal)	Perceived community environmental quality	H1	The higher the environmental quality perceived by residents, the more opportunities for positive contact with the outdoors are provided
Positive spatial contact opportunities (being away, fascination, compatibility, extent)	Restorative environmental perception	H1	Residents perceive restorative experiences through environmental quality, i.e., gaining positive contact.
Satisfaction of self‑existence needs (emotional relief, attention restoration)	Restorative environmental perception	H2	Restorative perception is the subjective evaluation of the degree of satisfaction of self‑existence needs.
The relationship between self-fulfillment and a sense of belonging through coexistence	Community identity	H2	Ongoing self-fulfillment is linked to a sense of community identity at the level of coexistence
Attributes of typical urban residents (age)	Age groups (children, young, middle‑aged, older adults)	H3	The structure of self-existence and co-existence needs varies across different age groups, and is linked to between-group differences in pathway strength

Based on the above mapping, the core logical chain of this study can be summarized as follows: typical human habitation (the perceived quality of the community environment) satisfies the self-existence needs of typical urban dwellers by providing opportunities for positive spatial contact (restorative environmental perception), which in turn is linked to emotional belonging (community identity) at the level of coexistence; the age characteristics of typical urban dwellers may be associated with between-group differences in this associative structure. The various pathways will be empirically tested using a structural equation model in the following sections.

It should be noted that this study did not directly measure the core constructs of the ‘Homo Urbanicus’ theory, such as ‘self-existence’, ‘coexistence’, ‘events’, ‘time’, ‘space’ and ‘opportunities for spatial contact’. In this study, the theory serves as an explanatory framework: it is used to provide a theoretically consistent interpretation of the observed patterns of association between variables, rather than as a mechanistic model subject to direct empirical testing. Specifically, this study treats the perception of a restorative environment as a proxy for ‘fulfilment of the need for self-existence’ and community identity as a proxy for ‘emotional belonging through coexistence’; this mapping constitutes an operationalization attempt at the level of theoretical interpretation. Whether a correspondence exists between the four elements and the observed variables cannot be directly determined from the current cross-sectional association data and awaits verification through the subsequent development of direct measurement tools.

### 2.2 Study sample

The data for this study were derived from a questionnaire survey of community residents conducted in Yangpu District, Shanghai, between November and December 2025. A two-stage purposive sampling design was employed. In the first stage, guided by the principle of maximizing diversity, 36 residential estates were selected from the 12 sub-districts of Yangpu District. The selection took into account factors such as the estates’ location, year of construction and environmental quality, ensuring that the sample estates covered as wide a range of residential community types within the area as possible.

In the second phase, residents were recruited within each housing estate using a convenience sampling method involving on-site approach. During the survey period, trained interviewers entered communal areas of the estates (such as estate entrances, central green spaces and fitness areas) to invite residents present to scan a QR code with their mobile phones, authorize access via WeChat and complete the questionnaire on the Wenjuanxing platform. To ensure coverage across all age groups, interviewers took care to recruit residents of different genders and age groups; for the children’s group (aged 0–17), a total of 108 children aged between 3 and 17 years (*M* = 10.50 years, SD = 3.19, median 11 years) actually completed the questionnaire, of whom only one was aged 3. Given the significant disparity in cognitive development and reading comprehension between very young children and school-age children, on-site staff who had undergone standardized training were arranged to provide one-to-one assistance during data collection: The staff strictly adhered to the principle of neutrality, limiting their role to explaining the meaning of each question and the options (for example, translating ‘sense of belonging’ into everyday expressions such as ‘do you like and miss this place?’), without interfering with the respondents’ subjective judgements or choice of options; for very young children who were not yet able to read independently, the interviewer read each question aloud and marked the appropriate option based on the child’s verbal response, to ensure that the children were able to complete the assessment based on their genuine feelings. Respondents in all other age groups (young adults, middle-aged adults and older adults) completed the questionnaire independently under the guidance of staff. The questionnaire was completed on an anonymous and voluntary basis, with respondents giving their informed consent prior to participation; young children completed the questionnaire in the presence of a parent or legal guardian. The interviewer explained the purpose of the study, the anonymous nature of the process and the principle of voluntariness to the guardian and obtained their verbal consent; the questionnaire was only commenced after the child’s own consent had also been obtained. The average time taken to complete the questionnaire was approximately 9 min.

A total of 1,154 questionnaires were collected for the survey. Two hundred twenty-five invalid questionnaires (19.5 per cent) were excluded based on the following criteria: (1) the location data of the respondents indicated that they were not within the 36 target residential estates (primarily because the questionnaire link had been forwarded and completed by residents outside the target estates); (2) Data quality issues were identified (e.g., responses completed in an unreasonably short time, or responses showing a pattern of regularity). A total of 929 valid questionnaires were ultimately obtained, representing a valid response rate of 80.5 per cent.

The 929 valid respondents were distributed across 36 residential estates, with the valid sample size per estate ranging from 2 to 61 individuals, averaging 25.8 per estate (median: 29). The sample exhibited a typical clustered (nested) structure at the estate level; the statistical treatment of this structure is described in Section 2.4. It should be noted that this study employed non-probability convenience sampling; whilst the sample covers different types of residential estates, it does not constitute a strictly probabilistic sample of Yangpu District residents, and caution should be exercised when generalizing the study’s conclusions to a wider population. The demographic characteristics of the sample are presented in Table 2.

**Table 2:** Demographic data for the sample.

Demographic data	*N*	%
Age		
Children	108	11.63
Young adults	149	16.04
Middle-aged adults	183	19.70
Older adults	489	52.63

### 2.3 Measurement of variables

#### 2.3.1 Dependent variable: community identity

The measure of community identity was adapted from the Neighbourhood Attachment Scale (NAS) developed by Bonaiuto et al. (1999). The original scale had a unidimensional structure comprising six items, primarily measuring residents’ positive emotional orientation towards their place of residence and their reluctance to move away. In this study, taking into account the context of Chinese urban communities, two core components measuring emotional attachment and reluctance to leave were selected from the original scale (‘I have a strong sense of belonging to this community and feel very at home here’ corresponds to the original item ‘Now this community is a part of me’; ‘If I had to move away, I would feel very reluctant’ corresponds to the original item “It would be very hard for me to leave this neighborhood”), and added one item reflecting local pride (“I feel very proud when others mention this community”), thereby forming a three-item abridged version; the items were drafted in Chinese and revised following discussions within the research team to align with the everyday language of community residents.

Community identity is measured across three dimensions:

I have a strong sense of belonging to this community; it feels very familiar to me.

If I had to move away, I would find it very hard to leave.

I feel very proud when others mention this community.All items are scored on a scale of 1 to 5: 1 = strongly disagree, 2 = somewhat disagree, 3 = neutral, 4 = somewhat agree, 5 = strongly agree. A higher score indicates a stronger sense of community identity among residents.

#### 2.3.2 Independent variable: perceived community environmental quality

This study defines ‘perceived community environmental quality’ as residents’ subjective assessment of their satisfaction with the overall environmental quality of their community, including green spaces, hygiene, facilities, safety and services, as measured through seven self-reported satisfaction items. It should be noted that this variable does not correspond to objective environmental indicators such as GIS-based built environment audits, green space ratios or environmental monitoring data; rather, it measures the environmental quality ‘as perceived’ by residents. Consequently, the term ‘perceived community environmental quality’ is used consistently throughout this paper, and the research conclusions should be understood as relating to environmental perceptions rather than objective environmental conditions. The seven-dimension items were developed with reference to the dimensional framework of the Perceived Residential Environment Quality (PREQ) questionnaire by Bonaiuto et al. (1999). This framework divides the perception of residential environment quality into four major domains—architectural and planning characteristics, social relations, service facilities, and environmental context—comprising a total of 11 specific content dimensions; In light of the specific circumstances of Chinese urban communities, this study selected seven aspects most directly relevant to residents’ daily lives—landscaping, environmental hygiene, pedestrian pathways, sports and exercise facilities, community safety, property management services, and noise and air pollution—measuring each aspect using a single satisfaction item, whilst adapting the original scale’s ‘agreement’ statements into a satisfaction rating format. The items were drafted in Chinese and, following discussion and revision by the research team, were used in the formal survey. Perceived community environmental quality comprises seven dimensions: ‘Landscaping’, ‘Environmental Hygiene and Cleanliness’, ‘Condition of Pedestrian Paths’, ‘Availability of Sports and Exercise Facilities’, ‘Sense of Community Safety’, ‘Property Management Services and Facility Maintenance’, and ‘Levels of Noise, Air Quality and Environmental Pollution’. All items are scored on a scale of 1 to 5: 1 = Very dissatisfied, 2 = Somewhat dissatisfied, 3 = Neutral, 4 = Somewhat satisfied, 5 = Very satisfied. A higher score indicates that residents rate the perceived quality of the community environment more highly.

#### 2.3.3 Mediating variables

Perceived restorativeness refers to the emotional relief, restoration of attention, sense of relaxation and environmental appeal experienced by residents within their community environment. Kaplan and Kaplan argue that a restorative environment must possess four characteristics simultaneously: ‘being away’, ‘fascination’, ‘extent’ and ‘compatibility’. The measurement of restorative environmental perception draws upon the Perceived Restorativeness Scale (PRS) developed by Hartig et al. (1997) and the Restorative Components Scale by Laumann et al. (2001), whilst drawing on the abbreviated paradigm of ‘single-item measures for each restorative component’ proposed by Berto (2005), which involves ‘measuring each restorative component with a single item’. Four items were developed in Chinese, tailored to everyday scenarios in Chinese urban communities: one item each for the components of being away, fascination, extent (expansiveness) and compatibility (e.g., ‘a space with no restrictions on activities’ corresponds to the ‘no restrictions on activities’ wording for the ‘extent’ component in the original scale). Following discussion and revision by the research team, the items were used in the formal survey; the applicability of PRS-type scales in the Chinese context has been validated (Wang et al., 2019).

Restorative environmental perception comprises four dimensions: ‘This place allows me to feel a sense of calm amidst high-pressure situations, far from the hustle and bustle and my worries’; “This place captures my attention and fascinates me’; ‘Here, I can blend into the surroundings with ease and do whatever I please’; ‘This is a space with no restrictions on activity, where I can do whatever I like to relax’. All items are scored on a scale of 1 to 5: 1 = strongly disagree, 2 = somewhat disagree, 3 = neutral, 4 = somewhat agree, 5 = strongly agree. A higher score indicates a stronger sense of restorative environmental perception.

#### 2.3.4 Control variables

This study incorporates gender, age, educational attainment, length of residence and monthly income into the conceptual model as control variables. Gender coding: 1 = male, 2 = female. Age groups: children (0–17 years), young adults (18–39 years), middle-aged adults (40–59 years) and older adults (60 years and over). The age range of 0–17 years was used as the criterion for grouping; the actual ages of the children included in the sample ranged from 3 to 17 years, with the specific distribution set out in Section 2.2. Educational attainment categories: 1 = below primary school level, 2 = primary school, 3 = lower secondary school, 4 = upper secondary school/vocational school, 5 = undergraduate/higher national diploma, 6 = postgraduate and above. Length of residence: 1 = 0–5 years, 2 = 5–10 years, 3 = 10 years or more.

It should be noted that the meaning of the control variable differs between the two groups: for adults aged 18 and over, educational attainment refers to the highest level of education they have completed; for child respondents under the age of 18, educational attainment refers to the stage of education they are currently enrolled in (primary school, lower secondary school or upper secondary school). As the meanings differ, this variable is used solely as a control variable within each group and is not used for cross-group comparisons. Income refers to the respondent’s own monthly income; there is also a semantic difference in the income variable between the two groups: for adults, income refers to the respondent’s own monthly income; for children, this item measures the amount of monthly pocket money at their disposal (answered by the children based on their own circumstances). The mean value of this variable in the children’s group is only 1.06, with virtually no variation within the group; therefore, whilst this variable is retained in the model for the children’s group, its coefficient is not interpreted.

### 2.4 Statistical analysis methods

This study employs descriptive statistical analysis and structural equation modelling (SEM) to investigate the complex relationships between perceived community environmental quality, perceptions of restorative environments, and community identity, as well as differences across different age groups. Structural equation modelling offers advantages in quantitative research involving multivariate interactions and between-group comparisons. The sample size for this study was 929, which constitutes a large sample; under these conditions, maximum likelihood estimation is robust to slight deviations in variable distributions. The 929 valid questionnaires selected for the study contained no missing values across all analyzed items; therefore, no additional processing of missing data was required.

In this study, the items measuring perceived community environmental quality, perceptions of restorative environments and community identity were all assessed using a 5-point Likert scale (1 = very dissatisfied – 5 = very satisfied). Given that Likert scales with five or more points are typically treated as continuous variables in structural equation modelling, this study treated the scores for each item as continuous observed variables in the model estimation (Rhemtulla et al., 2012).

Structural equation modelling was conducted using AMOS 29.0 software, employing the maximum likelihood estimation method. Prior to conducting cross-age-group comparisons, this study first tested measurement invariance (examining configural, metric and scalar invariance in sequence (Putnick and Bornstein, 2016; Cheung and Rensvold, 2002; Vandenberg and Lance, 2000)). Given that chi-squared tests are sensitive to minute differences in large samples, this study adopted ΔCFI ≤ 0.01 as the primary criterion (Cheung and Rensvold, 2002), whilst also reporting the *p*-values of the Δχ^2^ tests for reference. On the basis of established metric invariance, nested models were employed to formally test for inter-group differences in structural paths: using the freely estimated model as the baseline, equality constraints were applied across groups to the three paths—perceived community environmental quality → perception of restorative environment, perception of restorative environment → community identity, and perceived community environmental quality → community identity—respectively, with paired parameter difference tests (Critical Ratio) used to identify specific inter-group differences. The significance of indirect effects was determined by constructing 95% confidence intervals using the bias-corrected Bootstrap method (with 5,000 repetitions; Preacher and Hayes, 2008); an indirect effect was considered significant if the confidence interval did not include 0. Given that the respondents were nested within 36 neighborhoods and the design effects for all variables were greater than 1, this study further conducted a cluster-robust test for between-group differences in Mplus 8.3 using TYPE = COMPLEX and MLR estimation (with neighborhoods as the cluster units), and performed cross-validation using a reduced model with fewer free parameters than the number of clusters (a scale-means path model with 28 free parameters).

Given that the 929 respondents were nested within 36 residential estates, and that residents within the same estate may have interrelated environmental perceptions and psychological evaluations due to their shared built environment and community relationships, this study first examined the degree of clustering in the data. Based on a unidirectional random-effects variance model, the intraclass correlation coefficients (ICC(1)) were calculated: the ICCs for perceived community environmental quality, restorative environmental perception and community identity were 0.098, 0.138 and 0.106 respectively, all exceeding the empirical threshold of 0.05; The design effects (DEFF = 1 + (n̄ – 1) × ICC), calculated based on the average estate size (*n̄* = 25.8 people), were 3.43, 4.42 and 3.63 respectively, all significantly greater than 1. This indicates that ignoring clustering at the estate level would underestimate the standard error and consequently overestimate the level of statistical significance; therefore, the issue of clustering cannot be disregarded (Table 3).

**Table 3:** Intragroup correlation coefficients and design effects for each variable.

Variable	ICC(1)	DEFF
Perceived community environmental quality (PCEQ)	0.098	3.43
Restorative environmental perception (REP)	0.138	4.42
Community identity (CI)	0.106	3.63

ICC(1) is estimated based on a unidirectional random-effects variance component model; DEFF = 1 + (n̄−1) × ICC, where the average cluster size n̄ = 25.8. The DEFF values for all variables are significantly greater than 1, indicating that clustering cannot be ignored; therefore, cluster-robust standard errors are used.

To this end, this study re-estimated the entire structural equation model within a framework that accounts for community clustering: using community as the clustering variable (TYPE = COMPLEX), parameter estimates were obtained using Mplus 8.3 software with maximum likelihood robust estimation (ESTIMATOR = MLR), with standard errors calculated as sandwich-type cluster-robust estimators, and the model’s degrees of freedom were adjusted for finite sample size (Muthén and Satorra, 1995). This strategy retains the parameter estimates at the individual level whilst correcting for the impact of clustering on statistical inference; it is suitable for the situation in this study, where the number of clusters (36) is relatively limited and cluster sizes are uneven. The robust estimation results and their comparison with the original model are detailed in Sections 3.2 and 3.3.

In the structural model, perceived community environmental quality is treated as an exogenous latent variable that predicts perceptions of a restorative environment and community identity; perceptions of a restorative environment predict community identity; gender, age, educational attainment, length of residence and monthly income are treated as control variables, which predict community identity as exogenous observed variables, whilst allowing for unconstrained correlations between the control variables themselves and between the control variables and perceived community environmental quality. Consequently, all unidirectional arrows in the model point from the predictors to the dependent variable, whilst demographic variables are included in the model solely as covariates and are not predicted by any other variable.

To verify the suitability of the data for structural equation modelling, this study conducted a multi-factor confirmatory analysis of the measurement models within the conceptual model. The composite reliabilities of the three measurement models—perceived community environmental quality, perceived restorative environment, and community identity—were 0.881, 0.925 and 0.889 respectively, all exceeding the standard of 0.7. The average variance extracted (AVE) for the measurement models was 0.516, 0.756 and 0.728 respectively, all exceeding the standard of 0.5. The standardized factor loadings and reliability coefficients (SMC) for the observed variables were both greater than the widely accepted standards of 0.6 and 0.36, respectively. All measurement models demonstrated good reliability and validity and are suitable for structural equation modelling analysis (Table 4).

**Table 4:** Reliability and validity tests for the measurement model.

Confirmatory factor analysis	Observed variable	Standardized factor loading	Reliability coefficient	CR	AVE
Community environmental quality	ENV1	0.780	0.608	0.881	0.516
	ENV2	0.786	0.618		
	ENV3	0.775	0.601		
	ENV4	0.700	0.490		
	ENV5	0.604	0.365		
	ENV6	0.721	0.520		
	ENV7	0.638	0.407		
Restorative environmental perception	REC1	0.851	0.723	0.925	0.756
	REC2	0.859	0.738		
	REC3	0.896	0.803		
	REC4	0.871	0.759		
Community identity	IDEN1	0.865	0.748	0.889	0.728
	IDEN2	0.852	0.725		
	IDEN3	0.843	0.711		

The model fit results show that the overall model fit indices—GFI > 0.90, CFI > 0.90, AGFI > 0.90, IFI > 0.90 and RMSEA < 0.08—all meet the ideal criteria, indicating a good model fit. In the subgroup models, the GFI and AGFI were slightly below the strict ideal standard of 0.90, whilst the SRMR was slightly above 0.08; all were close to the critical values; however, the CFI, TLI and IFI all exceeded 0.90, whilst the RMSEA and χ^2^/df showed favorable results, suggesting that the aforementioned deviations represent minor fluctuations in individual indicators rather than overall poor fit. The structural validity of the subgroup model is generally acceptable and can support subsequent multi-group comparative analyses.

Furthermore, as the data in this study were all derived from subjective self-reports in the same questionnaire, the Harman single-factor test (Podsakoff et al., 2003) was employed. The results indicated that the first factor explained 25.3 per cent of the variance, which is below the critical threshold of 50 per cent. Confirmatory factor analysis was subsequently conducted, using a three-factor model as the baseline and comparing it with a single-factor model. The results showed that the three-factor model fitted the data well (χ^2^ = 313.748, df = 74, CFI = 0.971, RMSEA = 0.059), whilst the single-factor model fitted the data poorly (χ^2^ = 2502.837, df = 77, CFI = 0.708, RMSEA = 0.184); indicating that the three-factor model is significantly superior to the single-factor model, that there is good discriminant validity between the constructs, and that the data in this study do not exhibit serious common method bias.

## 3 Research findings

### 3.1 Descriptive statistical results

Based on data from 929 valid questionnaires, this study first conducted a descriptive statistical analysis of variables such as the demographic characteristics of the sample, the perceived quality of the community environment, perceptions of restorative environments, and community identity, in order to understand the distribution characteristics of the sample and the overall levels of each variable.

SPSS 27.0 was used to conduct a statistical analysis of the demographic characteristics of the community residents participating in the study. The results of the sample demographic analysis showed that, in this survey, 44.5 per cent of respondents were male and 55.5 per cent were female, indicating a relatively balanced gender distribution; the age structure encompassed children, young adults, middle-aged and older adults groups, with the middle-aged and older adults groups accounting for a relatively high proportion; in terms of educational attainment, the majority had completed senior secondary school or vocational training, or held a bachelor’s or associate degree, indicating an overall educational level that was slightly above average; more than half of the residents had lived in the estate for over 10 years, indicating a strong sense of community belonging; family structures were predominantly single-child and empty-nest households, with monthly incomes concentrated in the 5,000–10,000 yuan range, and the majority held Shanghai non-agricultural household registration.

The descriptive statistics for each variable indicate that the mean scores for perceived community environmental quality, restorative environmental perception and community identity are all at a slightly above-average level. The overall mean score for perceived community environmental quality was 3.66, indicating that residents generally rated the community environment favorably, although scores for dimensions such as property management services, facility maintenance and environmental hygiene were relatively low; the mean score for the perception of restorative environments was 3.79, reflecting that the community possesses a certain capacity to soothe emotions and promote relaxation and recovery; the mean score for community identity was 3.68, indicating that residents generally have a strong sense of belonging (see Table 5).

**Table 5:** Descriptive statistics for key variables.

**Variable name**	**Observed variable**	**Question content**	**Overall mean**	**Standard deviation (total)**	**Group Mean**			
					**Children**	**Young adults**	**Middle-aged adults**	**Older adults**
Community identity	IDEN1	I feel a strong sense of belonging to this community.	3.83	0.938	3.77	3.72	3.80	3.90
	IDEN2	I would feel reluctant to move away from this community.	3.65	1.136	3.65	3.53	3.62	3.71
	IDEN3	I feel proud when this community is mentioned.	3.57	1.062	3.38	3.43	3.63	3.63
Perceived community environmental quality	ENV1	How satisfied are you with the green spaces and landscaping within this community?	3.68	1.042	3.94	3.77	3.77	3.56
	ENV2	How satisfied are you with the environmental sanitation and cleanliness within this community?	3.54	1.055	3.54	3.60	3.75	3.44
	ENV3	How satisfied are you with the pedestrian walkways within this community?	3.67	0.987	3.82	3.62	3.75	3.63
	ENV4	How satisfied are you with the physical exercise facilities within this community?	3.57	1.060	3.81	3.57	3.63	3.50
	ENV5	How satisfied are you with the sense of safety within this community?	4.08	0.876	4.05	4.02	3.99	4.15
	ENV6	How satisfied are you with the property management services, community facilities, and environmental maintenance within this community?	3.45	1.096	3.70	3.49	3.70	3.28
	ENV7	How satisfied are you with the noise levels, air quality, and environmental pollution within this community?	3.66	1.027	3.69	3.56	3.69	3.67
Restorative environmental perception	REC1	This place offers a sense of being away from daily pressures and helps soothe my emotions.	3.81	0.839	3.82	3.74	3.81	3.82
	REC2	This place is sufficiently fascinating to capture and sustain my attention.	3.67	0.933	3.67	3.52	3.61	3.74
	REC3	This place provides a compatible setting where I can freely pursue my preferred activities.	3.87	0.868	3.89	3.75	3.85	3.90
	REC4	This place imposes no constraints on my activities, allowing me to engage in any relaxing pursuits.	3.80	0.882	3.81	3.69	3.77	3.84
Control variables	Gender	What is your gender?	1.56	0.497	1.44	1.55	1.65	1.55
	Age	What is your age?	54.24	23.017	10.50	32.21	48.61	72.72
	Education	What is your highest level of education attained?	3.83	1.307	2.30	5.18	4.39	3.55
	Length of residence	How long have you been living in this community?	2.31	0.854	1.69	1.78	2.25	2.64
	Monthly income	What is your approximate monthly income (in RMB)?	2.65	0.952	1.06	3.30	2.95	2.69

Furthermore, there were certain differences in the scores for each variable across different age groups: in terms of observed means, the children’s group gave relatively higher ratings for the perceived quality of the community environment, whilst the older adults group gave relatively lower ratings for certain environmental dimensions, such as property management services and environmental hygiene; scores for the perception of restorative environments were similar across all groups; community identity generally increases with age, being strongest among older adults and relatively weakest among young adults. The above differences between groups represent descriptive comparisons of observed means and have not undergone formal statistical testing; they provide a data foundation for subsequent structural equation modelling analysis.

### 3.2 Analysis of the full-sample model

When mediating variables are present in a model, the relationship between the independent and dependent variables should be decomposed into total, direct and indirect effects. Table 6 presents the model fit results for the full sample, whilst Tables 7, 8 present the effect decompositions and their Bootstrap confidence intervals; Figure 2 illustrates the full-sample model. The structural equation model for the full sample fits well overall, with all indicators meeting acceptable standards (see Table 6). Unless otherwise specified, the path coefficients reported in this study are standardized estimates (β); the results of the cluster-robust standard error checks for each path are presented in Table 9.

**Table 6:** Model fit statistics.

Model	**CFI**	**GFI**	**AGFI**	**IFI**	**RMSEA**	**χ** ^**2**^ **/df**	**TLI**	**SRMR**
Ideal standard	>0.90	>0.90	>0.90	>0.90	<0.08	<5	≥ 0.90	≤ 0.08
Overall model	0.962	0.947	0.928	0.962	0.052	3.550	0.953	0.042
Multi‑group model	0.944	0.893	0.854	0.945	0.032	1.942	0.931	0.089

**Table 7:** Total, direct and indirect effects of the model.

Independent variable	Mediating variable	Dependent variable		
	Restorative environmental perception	Community identity		
		Total effect	Direct effect	Indirect effect
Perceived community environmental quality	0.590***	0.598***	0.290***	0.308***
Restorative environmental perception	—	0.522***	0.522***	—

*** Means significant at the 1% significance level, ^**^ Means significant at the 5% significance level, ^*^ Means significant at the 10% significance level.

**Table 8:** Bootstrap test of the mediating effect of restorative environmental perception on the relationship between perceived community environmental quality and community identity.

Variable	Bootstrap			
	Bias-Corrected 95% CI		Percentile 95% CI	
	Lower	Upper	Lower	Upper
Perceived community environmental quality → Community identity	Total effect			
	0.532	0.661	0.533	0.661
	Direct effect			
	0.206	0.378	0.205	0.376
	Indirect effect			
	0.247	0.375	0.247	0.375

**Figure 2:**
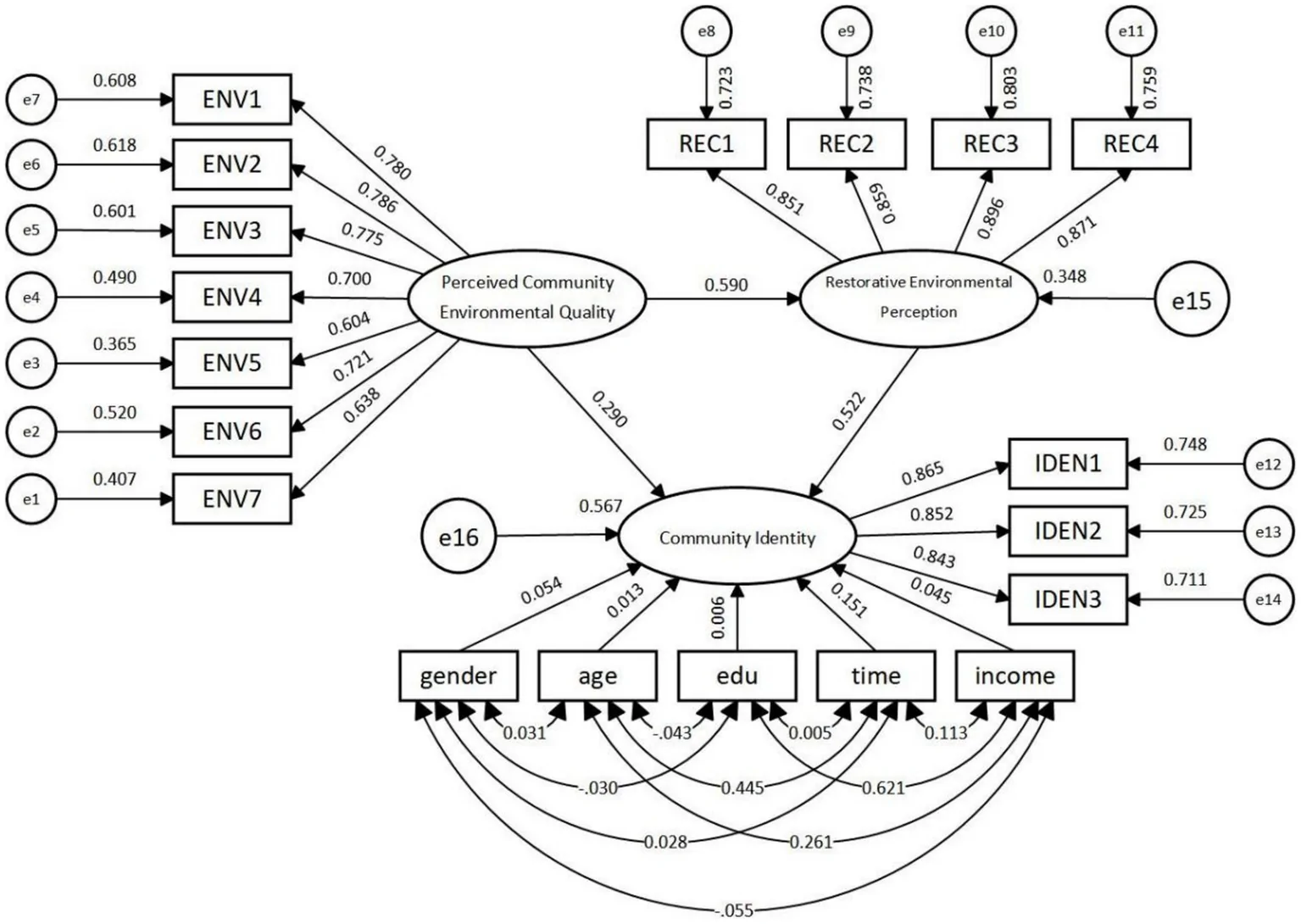
Overall sample model.

**Table 9:** Cluster-robust standard errors.

**Path**	**SE**
PCEQ → REP	0.042
REP → CI	0.069
PCEQ → CI(Directly)	0.062
Indirect effects	0.044

After controlling for demographic variables such as income, educational attainment, age, gender and length of residence, the total effect, direct effect and indirect effect (mediated by the perception of a restorative environment) of the perceived quality of the community environment on community identity were all significant at the 5 per cent level. The total effect was 0.598 (*p* < 0.001), the direct effect was 0.290 (*p* < 0.001), and the indirect mediating effect was 0.308 (*p* < 0.001), with the mediating effect accounting for 51.5 per cent. This indicates that perceived community environmental quality is directly associated with community identity and may also be indirectly linked to it via restorative environmental perception. In the chain mediation pathway ‘perceived community environmental quality → restorative environmental perception → community identity’, the indirect effect of restorative environmental perception was significant, indicating that restorative environmental perception plays a significant mediating role between the two; In particular, there is a significant positive correlation between perceived community environmental quality and restorative environmental perception (coefficient 0.590, *p* < 0.001), whilst the path coefficient between restorative environmental perception and community identity is 0.522 (*p* < 0.001), indicating a significant positive correlation between restorative environmental perception and community identity.

The 929 respondents were nested within 36 communities; the model was subsequently re-estimated in Mplus 8.3 with community as the cluster variable and using robust standard errors (MLR, TYPE = COMPLEX). The results showed that: Perceived community environmental quality → Perceived restorative environment (*β* = 0.596, SE = 0.042); Perceived restorative environment → community identity (*β* = 0.519, SE = 0.069); and the direct effect of Perceived community environmental quality on community identity (*β* = 0.286, SE = 0.062) and the indirect effect (*β* = 0.309, SE = 0.044) of perceived community environmental quality on community identity were both significant at the *p* < 0.001 level. The model fit was good (CFI = 0.958, RMSEA = 0.041, SRMR = 0.042), and the effect sizes were very similar to those in the original model, indicating that the research conclusions remain unchanged despite the community clustering.

### 3.3 Comparison of model differences across age groups

Using a structural equation multi-group analysis approach, the sample was divided into four groups—children, young adults, middle-aged adults and older adults—and a multi-group model was constructed for simultaneous estimation. Prior to conducting cross-group comparisons of structural paths, measurement invariance was first tested; on this basis, formal tests of inter-group differences in path effects were conducted using nested model equality-constraint tests and paired parameter comparisons (see Tables 10–13 and Figures 3–6).

**Table 10:** Results of the clustered model.

Independent variable		Mediating variable	Dependent variable: Community identity		
		Restorative environmental perception	Total effect	Direct effect	Indirect effect
Children	Perceived community environmental quality	0.666***	0.567***	0.239	0.328
	Restorative environmental perception	-	0.493***	0.493***	-
Young adults	Perceived community environmental quality	0.664***	0.702***	0.294***	0.408
	Restorative environmental perception	-	0.615***	0.615***	-
Middle-aged adults	Perceived community environmental quality	0.524***	0.553***	0.196**	0.357
	Restorative environmental perception	-	0.681***	0.681***	-
Older adults	Perceived community environmental quality	0.593***	0.612***	0.366***	0.246
	Restorative environmental perception	-	0.415***	0.415***	-

The coefficients in the table are standardized estimates; the significance of path coefficients, total effects and direct effects was determined using the within-group critical ratio (CR) test; for indirect effects, only point estimates (a × b) are reported, with their confidence intervals and significance levels shown in Table 13. ***p < 0.001, **p < 0.01, *p < 0.05 (two-tailed).

**Table 11:** Results of the invariance test for the four age groups.

Model	χ^2^	df	CFI	RMSEA	SRMR	Comparison of models	Δχ^2^	Δdf	P	ΔCFI
M1 Configural	651.511	296	0.959	0.036	0.060	–	–	–	–	–
M2 Metric	732.921	329	0.953	0.036	0.072	M2 vs. M1	81.410	33	<0.001	0.006
M3 Scalar	866.357	371	0.943	0.038	0.072	M3 vs. M2	133.436	42	<0.001	0.010

The criterion for significance is ΔCFI ≤ 0.01 (Cheung and Rensvold, 2002); Δχ^2^ refers to the nested model chi-squared difference test. ‘Multi-group model’ refers to the fit indices of the structural model that incorporates control variables and estimates the four groups separately (or simultaneously). M1 in Table 11 refers to the multi-group invariance test for the measurement model (excluding structural paths and control variables). As the model specifications differ, the fit statistics cannot be directly compared.

**Table 12:** Comparison of nested models with cross-group equality constraints on structural paths.

**Model**	**Content of the restrictions**	**χ** ^**2**^	**df**	**CFI**	**Δχ** ^**2**^	**Δdf**	***P***	**ΔCFI**
M4	Free estimate (baseline)	768.797	332	0.950	–	–	–	–
M5	PCEQ→REP Equivalent	776.275	335	0.949	7.478	3	0.058	0.001
M6	REP→CI Equivalent	776.370	335	0.949	7.573	3	0.056	0.001
M7	PCEQ→CI Equivalent	771.467	335	0.950	2.670	3	0.445	0.000
M8	The three paths are equivalent	783.721	341	0.949	14.924	9	0.092	0.001

Results of paired parameter comparisons (Critical Ratio): On the PCEQ → REP path, the children’s group scored higher than the middle-aged group (CR = −2.755, *p* = 0.006) and the older adults group (CR = −2.019, *p* = 0.043); on the REP → CI path, the middle-aged group scored higher than the older adults group (CR = −2.764, *p* = 0.006); there were no significant differences between groups on the direct PCEQ → CI path. The above tests were based on standard errors without cluster correction; The above tests were based on standard errors without cluster correction; the cluster-robust re-test is reported in Section 3.3. It should be noted that, under the setting incorporating control variables, the difference between the middle-aged and older adults groups on the direct path ‘Perceived Community Environmental Quality → Community Identity’ was −0.185 (*p* = 0.045); however, in the other two settings, the corresponding p-values were 0.138 and 0.147, respectively. Furthermore, the test for equal means across groups for this path did not reject the null hypothesis (M7, *p* = 0.445); therefore, this study does not regard this as substantive evidence of inter-group differences.

**Table 13:** Results of the bias-corrected bootstrap test for indirect effects across age groups.

**Age group**	**Indirect effects(a × b)**	**Lower limit of the 95% CI**	**Upper limit of the 95% CI**	***P***	**Direct effects *P***
Children	0.424	0.183	0.743	0.001	0.102
Young adults	0.318	0.223	0.459	<0.001	<0.001
Middle-aged adults	0.318	0.183	0.494	<0.001	0.002
Older adults	0.261	0.182	0.361	<0.001	<0.001

Bootstrap analysis was conducted using the bias-corrected method, with 5,000 bootstrap repetitions; a confidence interval that does not include 0 indicates that the indirect effect is significant. The figures in this table are derived from the multi-group free-parameter estimation model (M4) and differ slightly from those in Table 10 (separate estimates by group); this is to be expected. The two sets of figures are not interchangeable; the point estimates, confidence intervals and significance levels for indirect effects should be taken from this table.

**Figure 3:**
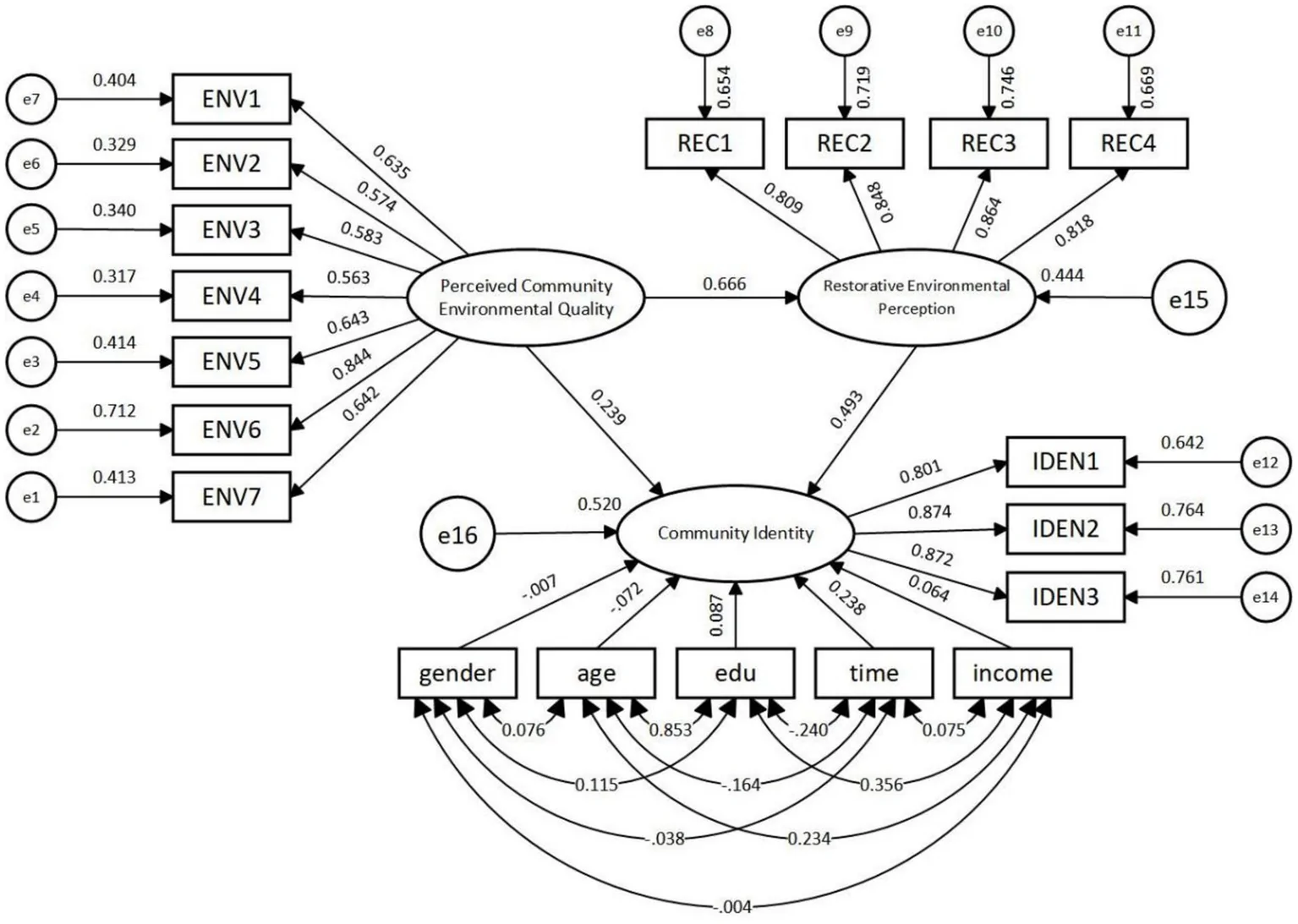
Children’s group.

**Figure 4:**
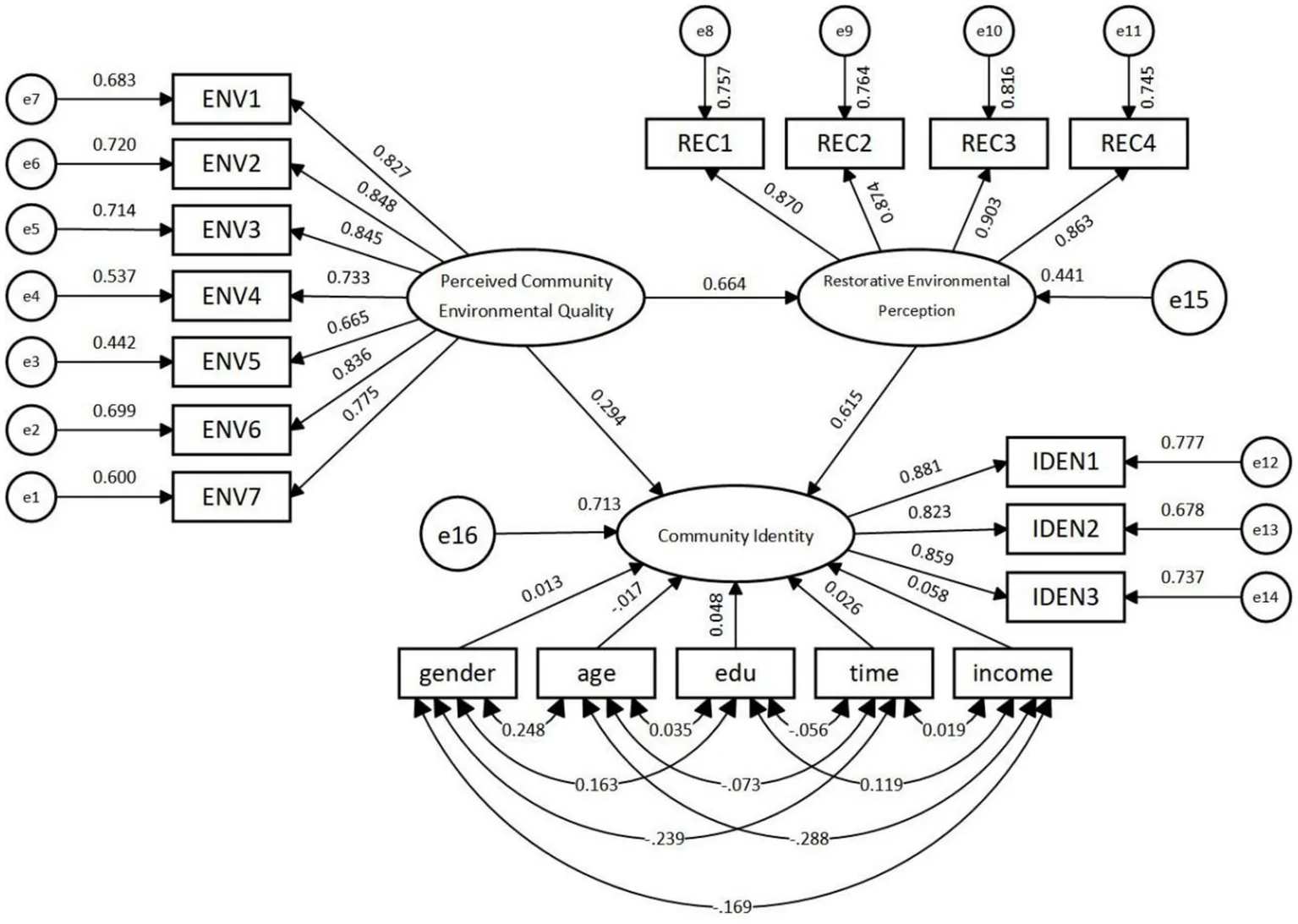
Young adults group.

**Figure 5:**
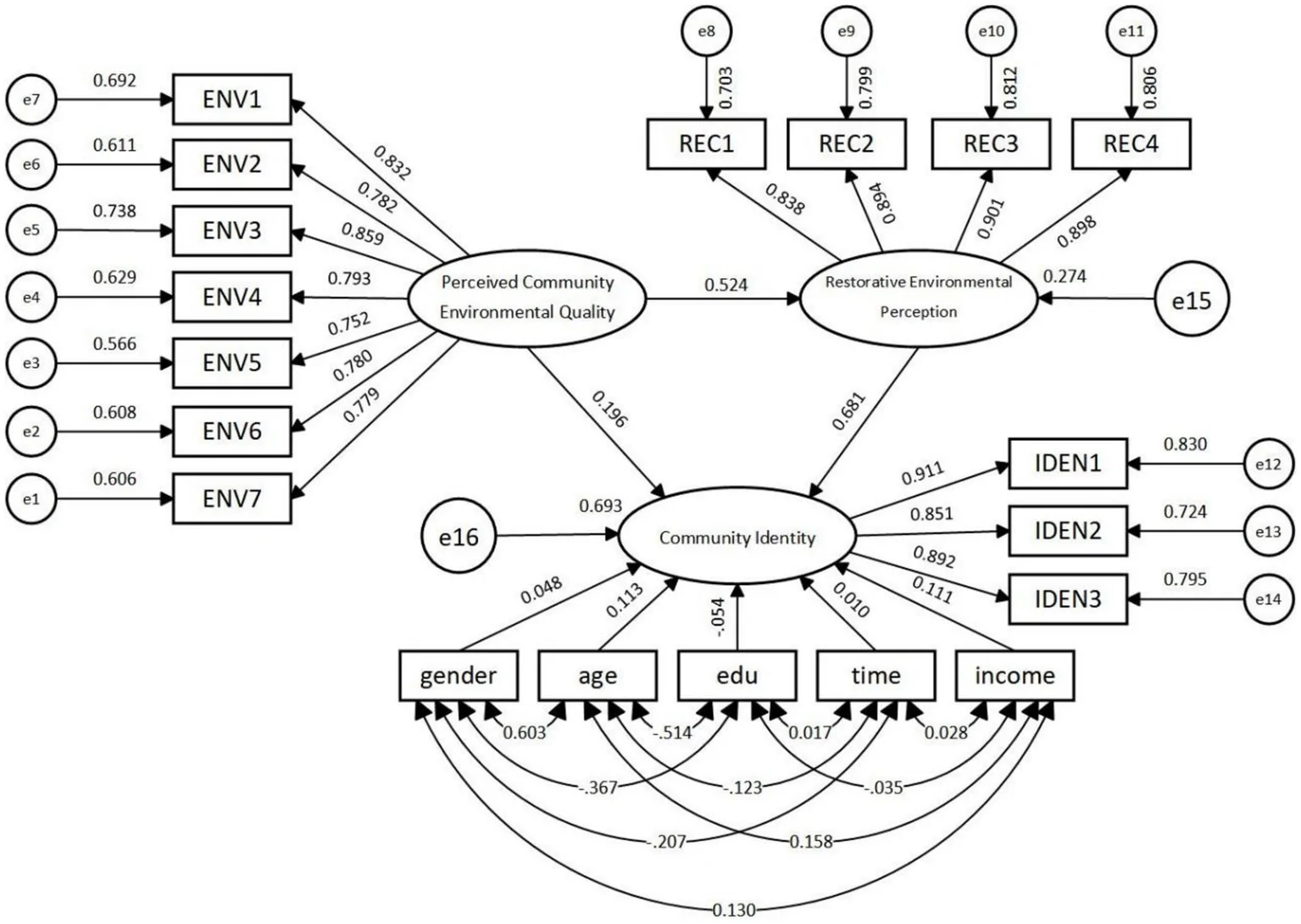
Middle-aged adults group.

**Figure 6:**
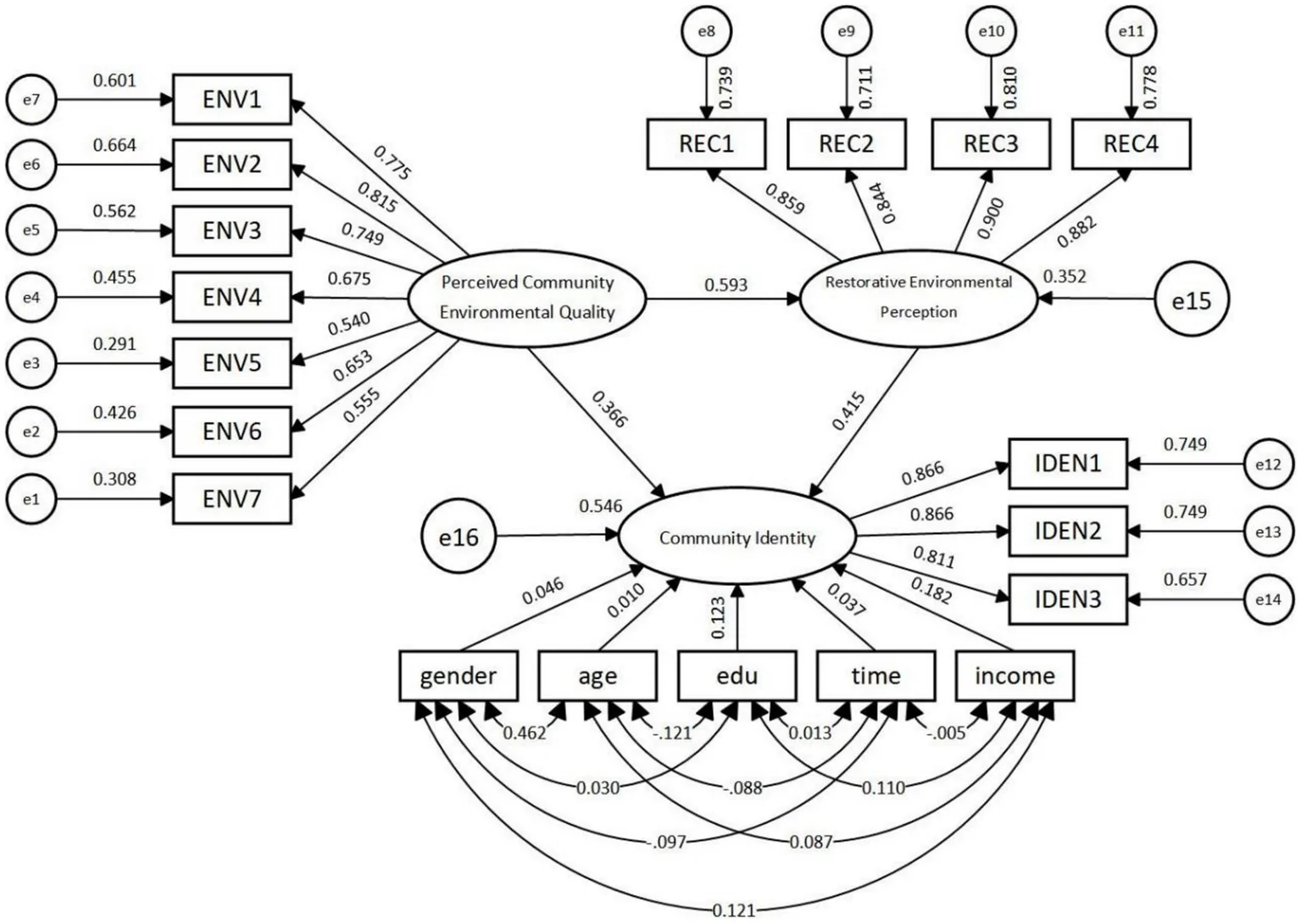
Older adults group.

It should be noted that this section involves three sets of estimation specifications, the values of which are not interchangeable: Table 10 presents segmented models estimated separately for each of the four age groups (including five demographic control variables), reporting standardized point estimates; Tables 12, 13 present equality-of-effects tests and bias-corrected Bootstrap tests for the multi-group model (estimated simultaneously across the four groups, excluding control variables); cross-group differences and the significance of effects are determined by these two tables; Furthermore, the results re-estimated using cluster-robust standard errors (MLR) at the end of this section are provided solely as robustness checks. Across all three sets of specifications, the children’s group consistently exhibits a ‘significant indirect effect and an insignificant direct effect’; the substantive conclusions of this study remain unchanged regardless of the specification chosen.

The results of the measurement invariance tests are shown in Table 11. The configural invariance model (M1) fits well, indicating that the four groups share the same factor structure. Compared with M1, the metric model (M2) did not exceed the decision criterion, confirming metric invariance; the scalar invariance model (M3) had a ΔCFI value at the boundary of the critical standard, suggesting that scalar invariance is broadly acceptable. The confirmation of metric invariance provides for subsequent cross-group comparisons of structural paths (Byrne et al., 1989).

The results of the tests for equivalence constraints on structural paths are presented in Table 12. Using the freely estimated model (M4) as the baseline, the application of equivalence constraints to each of the three paths (M5–M7) did not result in a significant deterioration in model fit; nor did the model fit deteriorate further when all three paths were constrained simultaneously (M8), indicating that differences in path coefficients across age groups are distributed locally. The above pairwise parameter comparisons revealed that, on the path from perceived community environmental quality to perceived restorative environment, the values for the children’s group were numerically higher than those for the middle-aged and older adult groups; on the path from perceived restorative environment to community identity, the middle-aged group scored higher than the older adult group; and there were no significant differences in the direct paths between any two groups. Given that the above pairwise tests were based on standard errors that were not adjusted for clustering, this study further re-examined the between-group differences using cluster-robust standard errors (Mplus 8.3, TYPE = COMPLEX, MLR, with neighborhoods as the cluster units; cross-validation was conducted using two sets of specifications: a multi-group model incorporating control variables and a scaled-mean reduction model with 28 free parameters). The results can be summarized in three points:

Firstly, on the path from Perceived Restorative Environment to Community Identity, the difference between the middle-aged and older adult groups was consistently significant across all three settings (differences of 0.270, 0.349 and 0.229, respectively; *p* = 0.021, 0.001 and 0.022). This was the only between-group difference in this study to receive cluster-robust support;

Second, on the path from perceived community environmental quality to restorative environmental perception, the numerical advantage of the children’s group did not consistently reach significance under cluster-robust standard errors—the *p*-values for the differences between the children’s group and the middle-aged group were 0.026, 0.025 and 0.059, whilst those for the differences with the older adults group were 0.147, 0.146 and 0.225. Therefore, this is reported as a descriptive pattern;

Thirdly, the between-group differences in direct and indirect effects were not significant under any of the three experimental designs (*p*-values for the four-group paired comparisons of indirect effects ranged from 0.251 to 0.635), although the indirect effects within each group were significant.

In the child sample, both perceived community environmental quality and perceptions of restorative environments were significantly and positively correlated with community identity, with total effect sizes of 0.567 and 0.493, respectively. The direct effect of perceived community environmental quality on community identity was not statistically significant, whilst its indirect effect was significant; It is worth noting that pairwise comparisons revealed that, in the children’s group, the path from perceived community environmental quality to restorative environmental perception was numerically higher than in the middle-aged and older adult groups; however, this difference did not consistently reach statistical significance in the cluster-robust test, and this study reports it as a descriptive pattern.

In the sample of young adults, both perceived community environmental quality and perceived restorative environment were significantly and positively correlated with community identity, with total effect sizes of 0.702 and 0.615, respectively. The direct effect of perceived community environmental quality on community identity was 0.294, whilst the indirect effect was 0.408; both were significant. In the separate estimates by subgroup, the indirect effect for the young adults group was relatively high among the four groups; However, it should be noted that in the multi-group Bootstrap estimates, the indirect effect value for the children’s group was, in fact, the highest. As the rankings from the two sets of estimates are inconsistent, this should be regarded as a descriptive pattern arising from sampling variation rather than a robust ranking.

In the sample of middle-aged adults, both perceived community environmental quality and restorative environmental perception were found to be significantly positively correlated with community identity, with total effect sizes of 0.553 and 0.681, respectively. The direct effect of perceived community environmental quality on community identity was 0.196, whilst the indirect effect was 0.357; both were significant. As the direct effect was relatively low, community identity amongst middle-aged residents appears to be achieved largely through the mediating pathway of restorative environmental perception; Pairwise comparisons revealed that the path from restorative environmental perception to community identity was significantly stronger in the middle-aged group than in the older adults group.

In the sample of older adults, both perceived community environmental quality and restorative environmental perception were significantly and positively correlated with community identity, with total effect sizes of 0.612 and 0.415, respectively. The direct effect of perceived community environmental quality on community identity was 0.366 and the indirect effect was 0.246; both were significant. In this group, the direct effect was relatively high, whilst the indirect effect was relatively low; the path from restorative environmental perception to community identity was significantly weaker than in the middle-aged group. Overall, community identity among older adult residents is relatively more directly associated with the community environment, whilst the mediating effect of perceptions of restorative environments is relatively weaker.

Taking into account the nested structure of the data, the subgroup models for the four age groups were similarly re-estimated using robust standard errors, with the residential community as the clustering variable (due to the limited sample size in each subgroup, the multi-group robust model did not include control variables, serving as evidence of the robustness of the patterns across groups). The multi-group cluster-robust model fitted well (CFI = 0.951, TLI = 0.950, RMSEA = 0.058 [0.050, 0.065], SRMR = 0.079), and the path patterns across groups were consistent with the original findings: in the children’s group, the direct effect of perceived community environmental quality on community identity was not significant (*β* = 0.215, SE = 0.174, *p* = 0.216), whilst the indirect effect was dominant (indirect effect = 0.348); in the young adults group (direct effect 0.306, *p* = 0.014; indirect effect 0.399), the middle-aged group (direct effect 0.185, *p* = 0.030; indirect effect 0.355) and the older adult group (direct effect 0.313, *p* < 0.001; indirect effect 0.282), all pathways remained significant. It should be noted that the test for cross-group equivalence constraints on the direct effect did not reject the null hypothesis (M7: Δχ^2^ = 2.670, Δdf = 3, *p* = 0.445, ΔCFI = 0.000; see Table 12). Consequently, this study reports the above results solely as findings within the children’s group.

The indirect effect of restorative environmental perception on community identity, mediated by the perceived quality of the community environment, was significant across all four age groups, with the confidence intervals for all four groups excluding 0 (see Table 13 for estimates and confidence intervals). With regard to direct effects, only the children’s group showed no statistical significance, whilst the effects were significant in the three groups comprising young adults, middle-aged adults and older adults. It should be emphasized that the lack of statistical significance in the direct effect does not imply that a direct association does not exist: the sample size for the children’s group was limited, resulting in insufficient statistical power for testing the direct effect, and the point estimate for the direct effect in this group was not zero. Therefore, this study reports only the evidence pattern that ‘the direct effect in the children’s group was not statistically significant, whilst the indirect effect was significant’; it does not infer from this that the perceived quality of the community environment ‘can only’ be associated with community identity via restorative perception.

In summary, the pathways linking the perceived quality of the community environment to community identity exhibit some age-related heterogeneity, but these differences are localised: indirect effects were significant across all four groups, and the mediating mechanisms were broadly comparable; The between-group differences supported by robust cluster tests were found in the ‘perception of a restorative environment → community identity’ pathway (the middle-aged group showed stronger effects than the older adults group, *p* = 0.021); on the ‘perceived quality of the community environment → perception of a restorative environment’ pathway, the numerical advantage observed in the children’s group did not reach statistical significance, and is therefore reported as a descriptive pattern.

## 4 Discussion

Building upon Professor Hok-Lin Leung’s Homo Urbanicus theory, this study employs an analytical framework comprising the four elements of ‘events, people, time and space’, whilst further introducing the philosophical dimension of the balance between self-existence and coexistence. Proceeding from the anthropological presupposition of the ‘Law of Nature’ (Liang, 2020), this study systematically elucidates the chain-like interrelationship between the perception of community environmental quality, restorative environmental perception and community identity. It reveals the underlying logic behind the heterogeneity of pathways across different age groups, thereby providing a theoretical basis for the planning of age-inclusive communities.

### 4.1 Overall association patterns: direct associations and indirect associations mediated by the restoration of sensory perception

#### 4.1.1 Test results regarding the positioning of the four elements—‘events, people, time and space’—and their interrelationships

In the context of this study, ‘events’ refers to the positive opportunities for spatial contact that residents seek within their communities—experiences such as ‘escaping the hustle and bustle’ and ‘blending into the environment’, as captured by restorative perception, and the sense of belonging and pride associated with community identity, are essentially two manifestations of the same spatial opportunity, progressing from functional fulfilment to emotional belonging. ‘People’ refers to community residents, who, as rational beings, are by nature characterized by both a concern for their own needs (self-existence) and a yearning for group belonging (coexistence). Professor Hok-Lin Leung distinguishes ‘time’ into the pre-event phase (the decision-making stage) and the post-event phase (the adaptation stage), emphasizing that repeated exposure during the post-event phase enables individuals to form stable, deep-seated evaluations of spatial opportunities (Liang, 2014). In this study, ‘time’ corresponds to the ‘post-event’ adaptation phase. Survey data show that 25.65 per cent of residents have lived in the community for 0–5 years, 17.59 per cent for 5–10 years, and 56.76 per cent for over 10 years. With over half of the residents having lived there for more than 10 years, the vast majority have entered a phase of continuous experience and evaluation of the community environment. It is precisely on the basis of this sample characteristic that this study uses this temporal cross-section as an observational window; this serves as the analytical focal point established by the researchers within the unified analytical framework of the ‘post-occupancy phase’, aiming to examine the nature of the relationship between restorative perception and community identity in a long-term residential context, rather than tracking diachronic changes within the same group. The concept of ‘space’ is defined as the community—the spatial scale most closely aligned with residents’ daily lives. Environmental factors such as green spaces, sanitation, facilities and safety are all within walking distance; their accessibility and carrying capacity directly influence the efficiency with which residents can access positive spatial experiences.

The results from the full sample are consistent in direction with the core propositions of the ‘Homo Urbanicus’ theory: the chain of associations between perceived community environmental quality, restorative environmental perception and community identity is significant (path coefficients of 0.590 and 0.522, *p* < 0.001), with indirect associations accounting for 51.5 per cent of the total association. At the interpretative level, this pattern of association may be understood as a possible manifestation of the balance between self-existence and coexistence at the community scale. It should be emphasized that, as self-existence, coexistence and the elements of ‘events, people, time and space’ were not directly measured, the consistency observed here merely indicates that the observed pattern of association aligns with the direction predicted by the theory; it cannot be regarded as empirical validation of the theory’s mechanisms. At the same time, these findings also resonate with Aristotle’s philosophy of human habitation, which states that ‘the city-state arises from the need for survival and is realized through the pursuit of happiness’ (Liang, 2014; Chen, 2017): as the fundamental unit of human habitation in contemporary cities, the primary function of a community is to provide residents with the spatial conditions necessary for ‘survival’ (safety, comfort, convenience and aesthetic appeal), thereby supporting the emotional sense of belonging that stems from the ‘pursuit of happiness’.

It should be noted that the explanation of the aforementioned mediating mechanism may also involve a reverse pathway: residents with a strong sense of community identity may offer more tolerant and positive evaluations of the community environment out of a sense of ‘loving the house and its surroundings’ or a reluctance to contradict themselves; furthermore, restorative environmental perception and community identity may be causally interrelated. The cross-sectional design of this study does not allow for the identification of the direction of these pathways; the above explanation of the mechanism should therefore be regarded as a candidate explanation consistent with theory rather than as a definitive conclusion.

#### 4.1.2 The ‘Homo Urbanicus’ theory as an interpretative framework: an analysis of the four elements of correlation patterns

The following analysis constitutes a theoretical deduction: the psychological processes in question are all theoretical hypotheses within the ‘Homo Urbanicus’ theoretical framework that require verification through longitudinal research, rather than conclusions that can be directly substantiated by the cross-sectional data of this study. Terms such as ‘transformation’ and ‘sublimation’ used in this paper should be understood in this context.

Firstly, ‘space’ constitutes the spatial prerequisite for this transition. The essence of human settlement lies in the pursuit of opportunities for spatial contact, and the key to such opportunities lies in accessibility. In this study, ‘space’ refers to the community—the spatial unit most closely aligned with residents’ daily lives—where environmental elements such as green spaces, sanitation, facilities and safety are all within walking distance of residents’ daily lives. It is precisely this appropriate scale that enables the perceived quality of the community environment to bypass the obstacles posed by insufficient accessibility at the urban scale, entering directly into the radius of residents’ perception and use. Only when the accessibility and carrying capacity of ‘space’ are sufficient do opportunities for spatial contact transform from an objective existence into something that can be accessed. Therefore, the appropriate scale of ‘space’ constitutes the primary condition for residents to effectively engage with positive opportunities for spatial contact; if there is a mismatch in spatial scale, even the highest environmental quality will struggle to translate into residents’ actual experiences.

‘Events’ encompass the objects and levels of transformation. Opportunities for spatial contact come in various forms, including positive and negative interactions. In this study, ‘events’ refer to the positive opportunities for spatial contact that residents seek within the community. Restorative experiences include emotional relief and renewed focus, a sense of ease and comfort in blending into the environment, and a feeling of freedom and relaxation unhindered by activity restrictions; these are closely linked to personal self-existence, embodying self-existence and fulfilling the need for self-existence; community identity, on the other hand, encompasses a sense of belonging, attachment and pride; it reflects an individual’s emotional connection to their group and their sense of place within it, embodying coexistence with others and fulfilling the need for coexistence. The spatial opportunities provided by perceived community environmental quality possess two attributes: the immediate restorative effect and the cumulative restorative effect. The immediate restorative effect refers to the momentary psychological benefits derived from a single encounter; the cumulative restorative effect, on the other hand, refers to the long-term psychological benefits resulting from repeated encounters, representing the continuous accumulation of fragmented experiences from individual encounters. The mediating effect was statistically significant and accounted for 51.5 per cent of the total effect, indicating that the indirect link via restorative environmental perception was only marginally larger than the direct link (0.308 versus 0.290); the two links are therefore best described as comparable in magnitude rather than as primary and secondary channels. This is the full extent of what the data from this study can establish. With regard to the relationship between the immediate restorative effect and the cumulative restorative effect, the ‘process’ is understood as the fulfilment of self-existence needs reflected in the immediate restorative experience, which, through the mediation of restorative environmental perception, ascends towards the sense of coexistence and belonging that community identity signifies; however, this ‘ascension’ is an explanatory inference proposed within the theoretical framework of ‘Homo Urbanicus’, rather than a statistical conclusion of this study. A clear distinction must be made between the epistemological statuses of the two: the proportion of the mediating effect indicates the relative weight of the indirect link within the total link, and it constitutes only directional support for this explanatory inference; since it has not been measured whether the progression from the fulfilment of self-existence needs to a sense of coexistence actually occurs, this proportion cannot be used as an indicator to measure the degree of this progression.

However, the objective conditions of ‘space’ and ‘events’ alone are insufficient to bring about transformation; the driving force behind transformation stems from human reason. Self-existence and coexistence with others are two inseparable and equally important aspects of human nature. The emotional relief and restoration of focus brought about by restorative experiences serve as direct evidence that the need for self-existence has been met; when residents consciously attribute this sense of fulfilment to the community environment, it naturally triggers a sense of community identity at the level of coexistence. Specifically, the fulfilment of the need for self-existence triggers a shift from self-existence to coexistence. The more an individual feels that ‘this environment is beneficial to me’, the more they tend to form a positive emotional evaluation of the group represented by that environment; the formation of a sense of coexistence, in turn, enhances the psychological value of the fulfilment of self-existence, and this sense of belonging elevates the significance of each restorative experience from ‘personal benefit’ at the individual level to ‘belonging here’ at the group level. The two are mutually dependent and reinforce one another, jointly constituting the intrinsic psychological driving force behind the transition from self-existence to coexistence.

However, the transition from self-existence to coexistence is not achieved through a single restorative experience; it depends on the accumulation of ‘time’. In this study, all residents were in the post-event phase, and more than half had lived in the community for over 10 years. This suggests that restorative perception is not a one-off psychological event, but rather a psychological resource that accumulates continuously through repeated daily interactions. The emotional relief experienced during every stroll and the restoration of focus during every brief rest are immediate manifestations of self-existence fulfilment; and the repeated occurrence and sustained attribution of this self-existence fulfilment over time enable residents to gradually integrate their fragmented individual experiences—such as ‘this community helps me to recover’—into a stable cognitive structure: ‘I have an emotional attachment to this community’. Without the accumulation of ‘time’, restorative perception would remain at the level of immediate emotional improvement, struggling to evolve into a deep and stable emotional attachment. The accumulation of time transforms quantitative change into qualitative change, and provides the psychological foundation for the sublimation of self-existence fulfilment into a sense of shared belonging.

In summary, within the interpretative framework of the ‘Homo Urbanicus’ theory, ‘space’ provides the prerequisite for engagement; ‘events’ embody the objects and levels of progression; ‘people’ impart the impetus and direction for transformation; and ‘time’ ensures the depth and stability of accumulation. These four elements are mutually interlinked and support one another. The accessibility of space determines whether residents can access spatial opportunities; the progressive nature of activities corresponds to the transition from self-existence to coexistence following such access; human rationality determines whether this transition can be achieved spontaneously; and the accumulation over time determines whether the resulting sense of belonging is stable and enduring. Under this interpretation, the chain of relationships between ‘environmental quality—restorative perception—community identity’ holds consistently across the entire sample, with indirect relationships accounting for 51.5 per cent of the total effect. As regards the statistical results themselves, this merely indicates that indirect links account for a significant proportion of the chain; however, interpreting this further as an expression of a tendency towards equilibrium between self-existence and coexistence, supported by the synergistic interaction of the four factors, constitutes an explanatory inference proposed by this study, the validity of which remains to be tested by research involving direct measurement of the aforementioned psychological processes.

### 4.2 An explanation of the mechanisms underlying path differences: a systematic explanation of path strength based on the four elements of ‘events, people, time and space’

Multigroup analysis revealed that indirect effects were significant across all four age groups, whilst direct effects were not significant only in the children’s group. Tests for equivalence constraints and paired parameter comparisons revealed that inter-group differences in path strengths were distributed locally: the path from ‘perception of a restorative environment’ to ‘community identity’ was stronger in the middle-aged group than in the older adults group, and this difference passed the cluster-robust test; the path from ‘perceived quality of the community environment’ to ‘perception of a restorative environment’ was numerically stronger in the children’s group, but did not pass the cluster-robust test; this study therefore reports it solely as a descriptive pattern; no significant inter-group differences were observed for the direct paths. Hok-Lin Leung’s framework of the four elements—‘events, people, time and space’—provides a unified theoretical basis for these differences.

#### 4.2.1 Path characteristics of the four age groups

1. Older adults: strong direct paths arising from the high integration of self-existence and coexistence.

The direct effect for the older adults group was 0.366, which was numerically relatively high among the four age groups, whilst the indirect effect was 0.246, which was numerically relatively low (descriptive comparison; formal tests revealed no significant differences in direct effects across groups, but the path from restorative perception to community identity was significantly weaker than that of the middle-aged group). The majority of older adult residents have lived in this community for several decades (Ju and Li, 2020; Han and Luo, 2020); their daily lives are deeply embedded within the community space, which serves not only as a physical dwelling but also as a repository of social networks and emotional memories. Long-term settlement has led the older adult population to form stable and direct emotional evaluations of their environment—the perceived quality of the environment directly triggers positive or negative emotional responses, with virtually no need for confirmation through the mediation of restorative experiences. For older adult residents, the incremental explanatory power of restorative perception as a mediating variable is relatively limited, thereby giving rise to a path model that relies more heavily on direct effects.

1. Young adults: a strong mediating pathway under the prominence of self-existence needs

The indirect effect for the young adult group was 0.408, the direct effect was 0.294, and within each group, the indirect effect was again found to be higher than the direct effect. Young adults are at the beginning or ascending stage of their careers (Zhao and Tang, 2021) and generally face the dual pressures of high work stress and long commutes; their daily lives are fast-paced and mentally draining. The community is regarded as a sanctuary from the pressures of daily life; young adults are highly sensitive to whether the community environment offers restorative perception, and every effective restorative experience directly addresses their most pressing psychological needs. At the same time, as young adults tend to reside in a community for a relatively short period, the link between their evaluation of the environment and deeper emotional attachments is still in the process of forming. The development of their sense of community identity remains largely dependent on continuous restorative experiences to validate and confirm it, thereby forming a mediation-dominated pathway with restorative experiences serving as the bridge.

1. Middle-aged adults group: the intermediary-dominated pathway under the implicit nature of self-existence needs

The direct effect for the middle-aged group was 0.196, the indirect effect was 0.357, and within each group, the structure showed that the indirect effect was greater than the direct effect. Members of the middle-aged group typically shoulder the dual responsibilities of being the family’s breadwinners and raising children; their daily lives are fragmented by multiple tasks such as commuting to work, children’s education and household chores, and their emotional responses to the community tend to be rationalized and functional. The attention of the middle-aged group is often focused not on the community itself, but on the broader stage of life beyond the community; consequently, their emotional evaluation of the community environment rarely translates into direct emotional projection. The association between the quality of the community environment and community identity is largely realized indirectly through restorative perception, exhibiting a path pattern dominated by indirect effects; moreover, the strength of the association along the ‘restorative environmental perception → community identity’ pathway is significantly higher than that observed in the older adults group.

1. Children: an intermediary-dominated pathway in the absence of differentiation between self-existence and coexistence

The indirect effect for the children’s group was 0.328, whilst the direct effect was not statistically significant. As no significant differences in the direct effect were detected across the four groups (see Table 12), this study does not interpret this as an absence of a direct association in the children’s group, but rather focuses on the established pattern of evidence across all groups: the children’s group exhibited a significant indirect effect, whilst the direct effect was not statistically significant; simultaneously, the path from perceived community environmental quality to restorative environmental perception in the children’s group was numerically higher than in the middle-aged and older adult groups (although this numerical advantage did not consistently reach statistical significance in the cluster-robust test; see Section 3.3 for details), suggesting that during childhood, the link between perceived environmental quality and restorative experiences is relatively strong. This is consistent with the structure of children’s needs: constrained by their level of cognitive development (Morgan, 2010; Pretty et al., 2003), children’s understanding of space relies more on concrete bodily sensations and experiential activities than on abstract environmental evaluations (Korpela et al., 2002; Wells and Evans, 2003), and their needs for self-existence and coexistence are highly dependent on specific outdoor play activities and immediate sensory experiences. Consequently, for children, the quality of the community environment is primarily manifested in the characteristics of places that are suitable for play and exploration and where immediate restorative sensations can be experienced; restorative experiences play a significant role in the formation of their emotional attachment to the community. It should be noted that the sample size for the children’s group was limited, and the accuracy of the relevant estimates requires further validation with a larger sample.

#### 4.2.2 Comparison of age heterogeneity from the perspective of the four elements

The differences in the pathways of the four age groups can be systematically compared across the four dimensions of ‘events, people, time and space’.

1. On the ‘events’ dimension, the strength of the association between exposure to the same positive spatial environment and psychological responses varied across different age groups. Formal tests revealed that the strength of the association in the ‘restorative environmental perception → community identity’ pathway was stronger in the middle-aged group than in the older adults group (CR = −2.764, *p* = 0.006; the cluster-robust test was significant across all three settings, *p* = 0.001–0.022); The path from perceived community environmental quality to restorative perception showed only a numerically superior effect in the children’s group; as the cluster-robust test did not consistently reach significance, this is reported as a descriptive pattern; there were no significant differences in the direct path between the groups. From a theoretical perspective, the self-existence needs of young adults may be in a relatively active and unsaturated state; consequently, the same level of positive exposure may yield more pronounced psychological benefits, and the association between restorative experiences and community identity is also closer. For middle-aged adults, the psychological response threshold to opportunities for spatial exposure may be relatively high, and the association between environmental quality and community identity is more often realized indirectly via restorative perception; community identity among older adults derives more directly from their overall evaluation of environmental quality, with a relatively weaker association with restorative experiences; the association between community identity and restorative experiences is particularly pronounced among children. It can thus be seen that the level of activation and saturation of the self-existence need at different life stages may help to explain the age-related differences in the strength of the association between opportunities for spatial contact and psychological responses.
2. On the ‘people’ dimension, this fundamental attribute of age is linked to structural differences in the needs for self-existence and coexistence. Among older adults, the need for coexistence has become highly internalized, whilst the need for self-existence tends towards saturation; this gives rise to a direct emotional evaluation model dominated by coexistence. Among young adults, the need for self-existence is temporarily more overt, serving as the primary driving force behind the pursuit of opportunities for spatial contact; this results in a mediated transformation model that begins with the fulfilment of self-existence. Among middle-aged adults, the need for self-existence tends to become implicit, and their emotional response to the community is relatively rational; for children, the needs for self-existence and coexistence have not yet diverged, and both develop simultaneously through direct experience. The relative importance of self-existence and coexistence varies across different life stages; within the explanatory framework, this structural difference corresponds to distinct patterns in the pathways linking environmental quality and community identity.
3. In terms of the ‘time’ dimension, differences in length of residence are linked to varying degrees of emotional attachment within the same post-event phase. Older adults, having lived in the same place for decades, have formed stable and direct emotional evaluations of their environment; in this context, time represents a process in which a connection is gradually established between environmental perception and stable emotional evaluation; young adults, having resided for a relatively short period, still need to verify whether the community meets their needs through ongoing restorative experiences; here, time plays the role of a waiting period during which experiences accumulate. The middle-aged adults group, with a duration of residence falling between the two extremes, has already established a certain foundation for emotional accumulation but has not yet reached the stage of direct internalization; consequently, they exhibit transitional characteristics characterized primarily by indirect pathways and supplemented by direct ones; although children have a duration of residence similar to that of young adults, their immature cognitive abilities prevent them from transforming the accumulation of time into stable emotional evaluations. These differences in the accumulation of time result in distinctly different patterns of direct and indirect path dependence among different age groups within the same post-event phase.
4. In the dimension of ‘space’, differences in the scale of activity are linked to the directness of environmental influence patterns. The scale of activity for older people and children is highly concentrated within the local community, yet similar scales correspond to markedly different patterns of association between the two groups.—Older adults, drawing on their mature cognition and long-term experience, form a more direct link between their sense of community belonging and their assessment of the environment; children, on the other hand, form a connection with their environment primarily through immediate experiences gained during specific play activities. It should be emphasized that this comparison is a descriptive interpretation: formal tests revealed no significant differences in direct effects across the four groups; therefore, it does not support the conclusion that ‘there is a direct effect for older adults but not for children’. The activity scale of middle-aged adults often extends beyond the boundaries of the community; the community is merely one of multiple spatial nodes. Their emotional evaluation of the community environment is easily influenced by external comparisons; within the group, the structure shows indirect effects to be stronger than direct effects, and the community, as a place of intermittent recovery, actually highlights its mediating role. Young adults also have a relatively broad scope of activity, but their high sensitivity to restorative environments effectively compensates for the lack of direct effects, ensuring that indirect effects remain dominant. The extent of their activity range does not directly determine the intensity of their sense of belonging, but rather indirectly moderates the pathway effects by influencing residents’ perceptions of the environment and their evaluation patterns.

#### 4.2.3 Summary

Based on a comprehensive analysis across the four dimensions of ‘events, people, time and space’, the differences in pathways among various age groups essentially reflect the varying combinations of self-existence and coexistence needs at different stages of life. Together, these four dimensions shape the distinct pathways through which different groups move towards a balance between self-existence and coexistence. There is no hierarchy of superiority or inferiority among these four pathways; their differences stem from the natural differentiation of the structure of needs at different life stages. The significance of community planning lies in identifying the genuine needs of various groups and, through the provision of differentiated restorative spaces, achieving a precise match between ‘typical urban dwellers’ and ‘typical human settlements’—only when the performance of the living environment responds to the needs structure of the Homo Urbanicus theory can the balance between self-existence and coexistence be truly realized on both the individual psychological and community spatial dimensions.

## 5 Conclusion

### 5.1 Summary of the study

The research findings indicate that residents of Yangpu District, Shanghai, rate the quality of their community environment, their perception of restorative environments and their sense of community belonging as generally above average. In terms of observed mean values, the children’s group gave relatively high ratings for perceived community environmental quality, whilst the older adults group gave relatively low ratings on certain environmental dimensions (such as property management services and environmental hygiene); scores for restorative environmental perception were similar across all groups; and community identity generally increased with age, being strongest among older adults and relatively weakest among young adults. The above differences between groups represent descriptive comparisons of observed mean values and have not been subjected to formal statistical testing.

At the macro-level, restorative environmental perception plays a significant mediating role between the perceived quality of the community environment and community identity: residents who perceive a higher quality of their community environment experience greater restorative effects and exhibit higher levels of community identity, with the relationship between these three variables following a pattern consistent with the mediating hypothesis. The mediating effect accounted for 51.5 per cent of the total effect, indicating that just over half of the association between perceived community environmental quality and community identity is mediated through restorative environmental perception. In other words, the link between environmental quality and community identity lies not merely in whether the environment is ‘good or bad’, but rather in whether residents ‘feel relaxed and restored’ within the community.

Comparisons across age groups further revealed that this overall mechanism exhibits both commonalities and localized differences across different life stages. Bootstrap tests indicated that the mediating role of restorative environmental perception held true across all four age groups; formal tests for between-group differences revealed that the differences were locally distributed: the association between the perception of a restorative environment and community identity was stronger in the middle-aged group than in the older adults group, and this difference was supported by cluster-robust tests; the association between the perceived quality of the community environment and the perception of a restorative environment was numerically stronger in the children’s group, but did not pass the cluster-robust test and is therefore reported as a descriptive pattern. Different age groups prioritize and combine the needs for ‘self-existence’ and ‘coexistence’ in different ways, resulting in distinct path patterns.

The above findings are consistent with the core hypothesis of this study: there is both a direct association and an indirect association—mediated by perceptions of restorative environments—between perceived community environmental quality and community identity; the relative strength of this indirect association shows some variation across different age groups. From an applied perspective, community regeneration should not merely aim to meet hard indicators such as green space ratios and the number of facilities; it should also provide restorative spaces tailored to the needs of different age groups within daily walking distance (Li, 2017; Zhong et al., 2022), enabling residents to gain restorative experiences through everyday use. Such experiences are consistently associated with enhanced community identity.

### 5.2 Design recommendations for community regeneration

Community regeneration should prioritize the creation of restorative soft features, such as quiet corners, rest areas and naturalized landscapes, rather than focusing solely on green space ratios and the number of facilities. For different age groups: older adults require an enhanced provision of quiet, safe everyday spaces within walking distance (Takano et al., 2002) to realize their direct benefits; for young adults, pocket parks and art trails that evoke a sense of ‘escapism’ and ‘charm’ should be created, utilizing their heightened sensitivity to facilitate restorative experiences; for middle-aged adults, emphasis should be placed on scenarios such as commuting routes and transitional spaces at entrances, where restorative experiences are accumulated through daily, unconscious exposure; and for children, play areas that combine safety, fun and opportunities for exploring nature should be provided, creating conditions for them to gain restorative experiences through hands-on play. Finally, the activity radii of different demographic groups should be identified to ensure the rational layout of restorative spaces (Xu and Yong, 2021; Zhang et al., 2024; Wan et al., 2025), and indicators measuring the compatibility between ‘typical urban dwellers’ and ‘typical living environments’ should be incorporated into community assessments to dynamically monitor residents’ satisfaction with spatial access opportunities and their perception of the balance between self-existence and coexistence.

### 5.3 Limitations of the study and future prospects

This study still has some limitations. Firstly, the sample was drawn exclusively from Yangpu District in Shanghai and was based on non-probability convenience sampling; consequently, its representativeness of the general population of Yangpu District is limited. In particular, working-age young and middle-aged adults and tenants may have been underestimated in the street intercept survey. The generalizability of the study’s conclusions to other cities, rural areas or groups from different cultural backgrounds requires further testing. Secondly, all variables in this study were derived from self-reports by the same cohort of respondents at a single point in time; the data can only reveal associations and indirect relationships between variables, and cannot confirm the direction of causality. In future research, a longitudinal tracking design or quasi-experimental methods could be adopted to test the direction and stability of the observed indirect relationships. Thirdly, this study did not directly measure the core constructs of the ‘Homo Urbanicus’ theory—namely ‘self-existence’, ‘co-existence’, ‘events’, ‘time’, ‘space’ and opportunities for spatial contact—but instead used perceptions of restorative environments and community identity as proxy indicators; consequently, the theory serves as an explanatory framework in this study rather than a mechanistic model that has undergone direct empirical testing. Future research could develop direct measurement tools for the aforementioned constructs to specifically test the theoretical mapping relationships. Fourthly, all core variables in this study were based on residents’ self-reports, which may be subject to common-method bias; furthermore, evaluations may have been influenced by the respondents’ emotional state at the time of completion. Although the comparison between Harman’s one-factor test and confirmatory factor analysis did not reveal any serious common-method bias, systematic bias may still exist between subjective perceptions and objective environmental conditions. Future research could integrate multi-source data, such as behavioral observations and physiological indicators, and incorporate objective environmental indicators—including green space coverage, noise levels and accessibility to public facilities—to examine the consistency between subjective perceptions and the objective environment, as well as their differential predictive power for psychological outcomes. Fifth, the ‘balance between self-existence and coexistence’ in the ‘Homo Urbanicus’ theory is a dynamic process; this study only characterized the mediating effect at a single point in time and failed to capture the long-term evolution of this state of balance. Sixth, the sample size for the children’s group was relatively limited (*n* = 108); future research should expand the sample of children to verify the robustness of the path patterns in this group. Seventh, although cluster-robust standard errors were employed in statistical inference to account for the nested structure at the community level, this strategy merely corrected the estimates of standard errors without modelling the underlying mechanisms at the community level. Overall, future research could expand the scope of the sample to include different types of cities, utilize multi-time-point longitudinal data, and incorporate additional indicators of living environment quality—such as aesthetic appeal and social vibrancy—to deepen our understanding of the ‘typical urban dweller–typical living environment’ match based on direct measurements.

## Data Availability

The original contributions presented in the study are included in the article/supplementary material, further inquiries can be directed to the corresponding author/s.
